# Abiotic drivers of the space use and activity of gray reef sharks *Carcharhinus amblyrhynchos* in a dynamic tidal environment

**DOI:** 10.1111/jfb.15825

**Published:** 2024-05-29

**Authors:** Anaïs Laurioux, Charlie Huveneers, Yannis Papastamatiou, Serge Planes, Laurent Ballesta, Johann Mourier

**Affiliations:** ^1^ MARBEC, Univ Montpellier, CNRS, IFREMER, IRD Sète France; ^2^ College of Science and Engineering Flinders University Bedford Park South Australia Australia; ^3^ Institute of the Environment, Department of Biological Sciences Florida International University North Miami Florida USA; ^4^ PSL Research University EPHE‐UPVD‐CNRS, UAR 3278 CRIOBE, Université de Perpignan Perpignan Cedex France; ^5^ Andromède Océanologie Carnon France

**Keywords:** accelerometer, acoustic telemetry, behavior, energyscape, foraging strategies, predator

## Abstract

Predators display rhythms in behavior and habitat use, often with the goal of maximizing foraging success. The underlying mechanisms behind these rhythms are generally linked to abiotic conditions related to diel, lunar, or seasonal cycles. To understand their effects on the space use, activity, and swimming depth of gray reef sharks (*Carcharhinus amblyrhynchos*), we tagged 38 individuals with depth and accelerometer sensors in a French Polynesian atoll channel exposed to strong tidal flow, and monitored them over a year. *C. amblyrhynchos* used a larger space during nighttime and were more active at night and during outgoing currents. Shark activity also peaked during the full and new moons. The swimming depth of sharks was mostly influenced by diel cycles, with sharks swimming deeper during the day compared to nighttime. The dynamic energyscape may promote the emergence of discrete behavioral strategies in reef sharks that use the south channel of Fakarava for resting and foraging purposes. Turbulence imposed by outgoing tides induces additional foraging cost on sharks, shifting their hunting areas to the southern part of the channel, where turbulence is less pronounced. Understanding when and where sharks are active and foraging is important for our understanding of predator–prey dynamics and ecosystem dynamics. This study highlights how abiotic rhythms in a highly dynamic environment likely generate spatiotemporal heterogeneity in the distribution of predation pressure.

## 1 INTRODUCTION

Biological rhythms play a fundamental role in animals, governing various aspects of their behavior and physiology (Rusak & Zucker, 1975). These rhythms are influenced by a combination of biotic and abiotic (e.g., environmental) factors. Changes in environmental conditions through natural cycles (e.g., diel, tidal, seasonal) can shape biological rhythms and habitat quality, and therefore movement and activity patterns (Ito et al., 2022; Railsback et al., 2015). For example, seasonal changes (e.g., photoperiod, temperature, sea ice melting) can induce the start of population‐wide events like smoltification of salmons (*Salmo gairdneri*, Richardson 1836) or migratory movements of aquatic animals (Andrews‐Goff et al., 2018; Wagner, 1974), while diel and tidal cycles can affect home range and habitat use in lingcod (*Ophiodon elongatus*, Girard 1854) (Tolimieri et al., 2009). Abiotic cycles in particular can influence the behavior and distribution of predators, potentially modulating their interaction with lower trophic levels and the structure of ecosystems. Furthermore, temporal and spatial heterogeneity in predation pressure can interact to create spatiotemporal dynamic landscapes of fears (Palmer et al., 2022), in which prey need to adjust their behavior to when and where predators are hunting. Variations in environmental conditions can also modify energy landscapes, affecting the cost of transport or the energy required to move and forage for predators and prey (Shepard & Lambertucci, 2013). Therefore, the influence of abiotic cycles on dynamic energy landscapes can regulate the foraging abilities of predators and govern the spatial distribution of behaviors of predators and prey (Papastamatiou et al., 2023), and structure trophic interactions in ecosystems.

Sharks are important predators in marine ecosystems and may play a role in structuring ecosystems, exerting important consumptive and non‐consumptive pressures on lower trophic levels (Heithaus et al., 2022). The predatory behavior of sharks and their activity is often influenced by the diel cycle, with most tropical species showing crepuscular or nocturnal peaks in activity, especially a few hours after sunset (Brewster et al., 2018; Kadar et al., 2019; Papastamatiou et al., 2015, 2018; Shipley et al., 2018). These evening peaks are likely due to opportune foraging times because of the physiological and anatomical (e.g., vision) advantages sharks have during these periods (Ito et al., 2022; Papastamatiou et al., 2015). Shark activity can also change over tidal stages (Byrnes et al., 2021; Lea et al., 2020), with sharks generally taking advantage of high tide to forage on tidal flats that are not available otherwise (Ackerman et al., 2000; Lea et al., 2020). In channels or regions with strong tidal currents, tides may directly change shark activity patterns as part of their dynamic energy landscape (McInturf et al., 2019; Papastamatiou et al., 2021). As increased activity in sharks is generally related to foraging, understanding when and where sharks are active and foraging is important for our understanding of predator–prey dynamics and ecosystem dynamics.

The gray reef shark (*Carcharhinus amblyrhynchos*, Bleeker 1856) population in the South Pass of Fakarava, French Polynesia, is one of the largest reef shark aggregations, with up to ~700 individuals within 17 ha (Mourier et al., 2016). *C. amblyrhynchos* partially sustain themselves on spawning fishes that move into the channel, including over 17,000 camouflage groupers (*Epinephelus polyphekadion*, Bleeker 1849) that congregate in the channel 2 weeks before the full moons between June and July (Mourier et al., 2016, 2019). *C. amblyrhynchos* hunt reef fishes at night, with minimal hunting during the day when sharks select regions of the channel with updrafts generated from tidal currents, enabling sharks to reduce their swimming activity and therefore metabolic costs (Labourgade et al., 2020; Mourier et al., 2016; Papastamatiou et al., 2021). While the influence of tidal currents on shark activity in Fakarava has been investigated during the day, less is known about the interaction between tidal and diel phases on the activity of *C. amblyrhynchos* and whether it can influence shark distribution and predation pressure in the channel.

Here, we determine the effects of tidal, diel, and lunar cycles on *C. amblyrhynchos* space use, swimming depth, and activity using acoustic telemetry. We predict that (1) space use and activity will increase at night due to increased nocturnal hunting, (2) activity and depth will be higher (and shallower) during the outgoing tide, regardless of time of day, due to sharks encountering strong turbulence during the ebbing tide (Papastamatiou et al., 2021), and (3) activity will be higher during full and new moons when current strength (and associated turbulence) is higher.

## 2 MATERIALS AND METHODS

### 2.1 Ethics statement

This study was conducted under the authority of the Direction de environment de Polynésie française under the convention 4469/MCE/ENV signed on 11 June 2017. Shark tagging was approved by FIU IACUC #200787.

### 2.2 Study site

**FIGURE 1:**
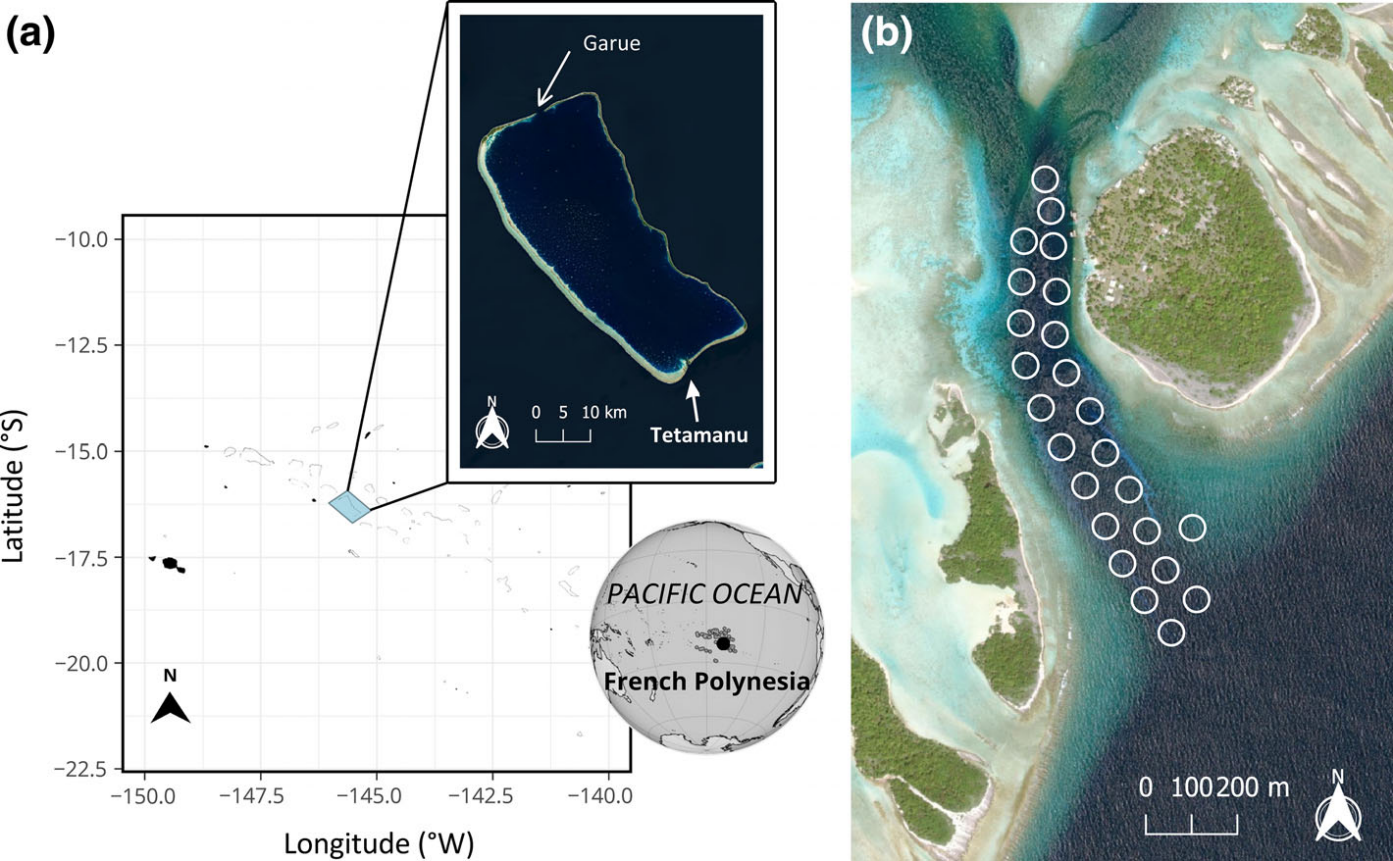
(a) Location of the south pass of Fakarava (Tetamanu) and (b) the acoustic array (circles) deployed inside the channel.

Fakarava Atoll is in the Tuamotu Archipelago, French Polynesia (16°30′ S, 145°27′ W). Two channels connect the ocean to the lagoon through its northern and southern parts (Figure 1a). We conducted the study in the southern pass, which has a main channel that branches off into two smaller channels on the lagoon side (Figure 1b). The main channel covers an area of 0.2 km^2^, with a maximum depth of 30–35 m, reaching 50 m at the entrance.

Tidal amplitude in French Polynesia is small, ~35 cm (Figure S1), but sufficient to generate strong currents because the connection between the lagoon of this large atoll and the ocean is limited to two passes. During incoming tides (flood tides), the current flows from south to north, resulting in updraft currents where the seabed rises towards the north (Papastamatiou et al., 2021) (Figure S2‐3). Conversely, outgoing tides (ebbing tides) are characterized by turbulent flows (Papastamatiou et al., 2021).

### 2.3 Acoustic telemetry

We captured and tagged 38 *C. amblyrhynchos* individuals in the main channel in June 2017. Divers caught sharks underwater at night by grabbing their tails and putting them into tonic immobility (Kessel & Hussey, 2015) to pass a looped rope around the tail. The shark was then brought to the surface and restrained upside down alongside the boat. We measured the total length of each shark to the nearest centimeter and made an incision on the ventral surface to implant a V13AP acoustic transmitter (Innovasea). The incision was closed using surgical staples (staple width 6.5 × 4.7 mm) from a Weck Visistat® 35 W Skin Stapler and the shark was released.

Acoustic tags were equipped with acceleration sensors, which record measurements of acceleration (hereafter referred to as activity) using a triaxial accelerometer. Tags sampled at 5 Hz for 20 s every 100–160 s for the first 120 days after which transmission delays changed to 50–110 s. A longer delay in the first 120 days was used to limit potential transmission collisions in the first 4 months because (1) sharks are more abundant during the grouper spawning aggregation when tagging was conducted and (2) some individuals are expected to disperse over time, allowing for a shorter transmission delay. The static contribution from gravity was filtered out, and the onboard microprocessor calculated dynamic acceleration using the root mean square value of all three axes (activity = √[*X* ^2^ + *Y* ^2^ + *Z* ^2^], averaged over time). The transmitters also included a pressure sensor to measure swimming depth with resolution of 0.1 m. Acceleration (range ± 4.9 m s^−2^, resolution 0.02 m s^−2^) was sampled at a 4:1 ratio with the pressure value.

V13AP tags transmit data at 69 kHz which were detected by an array of acoustic receivers (VR2W; Innovasea) covering a monitoring area of ~0.17 km^2^. We tested the performance of the acoustic receivers prior to deploying them. Detection range varied between day and night, with a 50% detection rate obtained at 100 m during the day that decreased to ~50 m at night (Figure S4). Consequently, we deployed 24 receivers in the main channel 50 m apart, enabling the detection of sharks across the entire channel during the day and night.

### 2.4 Activity space and repetitive space use analyses

Total (95%) and core activity (50% kernel utilization density [KUD]) spaces were calculated using the VTrack package, based on the estimated center of activity (COA) positions at regular 30‐min intervals and with a 50‐m smoothing factor (Simpfendorfer et al., 2002; Udyawer et al., 2018). Using the *HRSummary()* function, we calculated the kernel utilization density for all sharks combined over a year to represent space use at a population level. The space use of each shark was not estimated if fewer than five positions were available (Udyawer et al., 2018), which led to one shark being removed from further analyses. We calculated the total and core space use of each shark by month according to diel and tidal cycles to test their effect using linear mixed models (LMMs), with shark identity (ID) and month as random effects, using the *lmer* function from the lme4 package (Bates et al., 2015). The ‘Diel’ factor represents the daytime and nighttime periods that we determined based on daily sunset and sunrise times using the R package suncalc. The ‘Tide’ factor represents flood (incoming current in the channel) and ebbing (outgoing current) tides based on the Service hydrographique et océanographique de la marine (https://data.shom.fr/), a database of marine physical environment parameters. We determined the most suitable transformation and error distribution for each analysis by assessing the distribution of the response variables. All model combinations of fixed‐effect terms were run using the *dredge()* function (package MuMIn), with our full model being:

$$\mathrm{KUD}\;50/95\sim \text{Diel}\times \text{Tide}+{\left(\mathrm{ID}\right)}_{\mathrm{rand}}+{\left(\text{Month}\right)}_{\mathrm{rand}}.$$

We used Akaike's information criterion for small sample sizes (AIC_c_) to test relative support for each of the models (Burnham & Anderson, 2004), where AIC_c_ weight (wAIC_c_) is equivalent to relative model probability. We calculated the conditional (*R*_c_) and marginal (*R*_m_) *R* ^2^ for the mixed‐effect models (Nakagawa & Schielzeth, 2013) to estimate the proportion of variance among fixed and random effects combined and fixed effects alone, respectively. The residuals of the top‐ranked models were visually examined before the results were interpreted.

As June and July correspond to a period of massive spawning aggregation of camouflage groupers *E. polyphekadion* (Mourier et al., 2016) that could affect the behaviors of sharks, we also conducted the same analysis on a subset of the data after the months of June and July were removed.

### 2.5 Activity and depth

Duplicated detections (i.e., when a signal is detected by two receivers, which accounted for 6% of all detections) were removed from analysis. Activity and depth values were then averaged over 1 h to reduce the amount of raw data and improve calculation time. We constructed generalized additive mixed models (GAMMs) to test for the effects of diel, tidal, and lunar cycles on activity and depth using the mgcv package (Wood, 2011). Activity data were log‐transformed to correct the skewedness of residuals. An autoregressive process of order 1, corAR1 (Zuur et al., 2009) was also added to the random structure of the models to account for temporal autocorrelation. We checked the models for concurvity and selected the number of nodes using *gam.check*, which determines if the smoothing factor has been set too low. As with activity space, we compared each model using the Akaike's information criterion for small sample sizes (AIC_c_), the AIC_c_ weight (wAIC_c_), and conditional (*R*_c_) and marginal (*R*_m_) *R* ^2^ for the mixed‐effect models. The residuals of the top‐ranked models were visually examined before the results were interpreted.

As for the KUD analyses, we also conducted the analyses on acceleration and depth on a subset of the data after the months of June and July were removed.

We identified areas of high and low activity using COA positions and the Expectation–Maximization algorithm for Mixtures of Univariate Normals from the mixtools package (Benaglia et al., 2010). A distribution model was fit to the acceleration data (i.e., a two‐component mixture in which the standard deviations are assumed equal). Activity was thus classified into high and low groups based on the distribution of activity values (Figure S5). When the posterior probability that an acceleration value associated with one of these groups was greater than 0.75, COA positions of the acceleration value were classified in the corresponding group. We then estimated and plotted the KUDs of high and low activity values.

## 3 RESULTS

### 3.1 Activity space

The movements of 38 sharks (mean ± standard deviation 141 ± 10 cm total length, 34 females and four males) were tracked from July 2017 to the end of June 2018. This shark aggregation was characterized by a female‐biased sex ratio (F:M = 4:1; Mourier et al., 2016), explaining the greater number of females tagged. Most sharks were resident, with a residency index (i.e., number of days detected divided by the number of days at liberty) of 0.85 ± 0.23 and 29 sharks (76%) had a residency index >0.9 (Figure S6 and Table S1).

**FIGURE 2:**
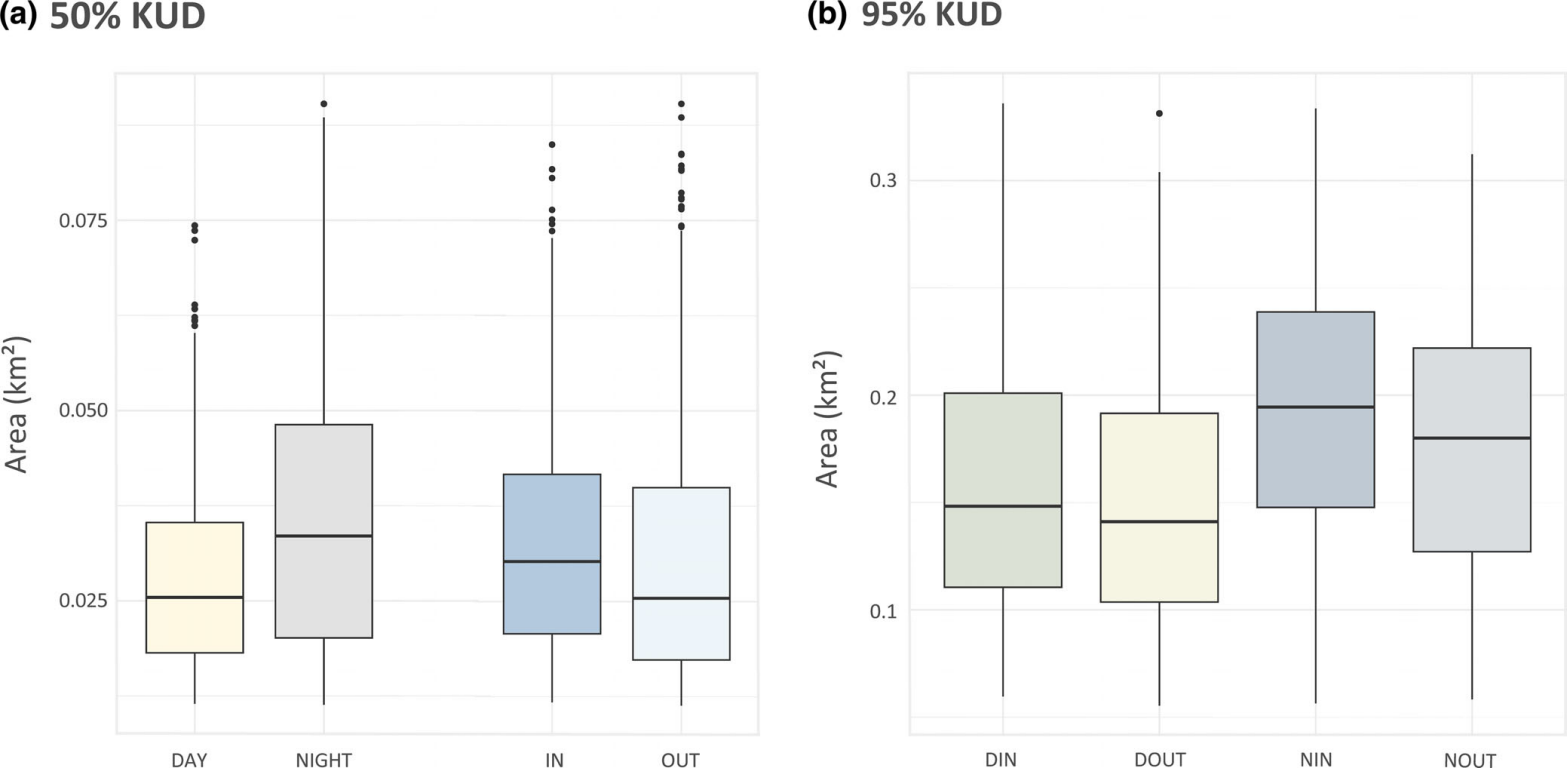
Effect of the diel and tidal cycle on the size of (a) 50% and (b) 95% kernel utilization density (KUD) areas by *Carcharhinus amblyrhynchos*. DIN, incoming current at daytime; DOUT, outgoing current at daytime; NIN, incoming current at nighttime; NOUT, outgoing current at nighttime.

**FIGURE 3:**
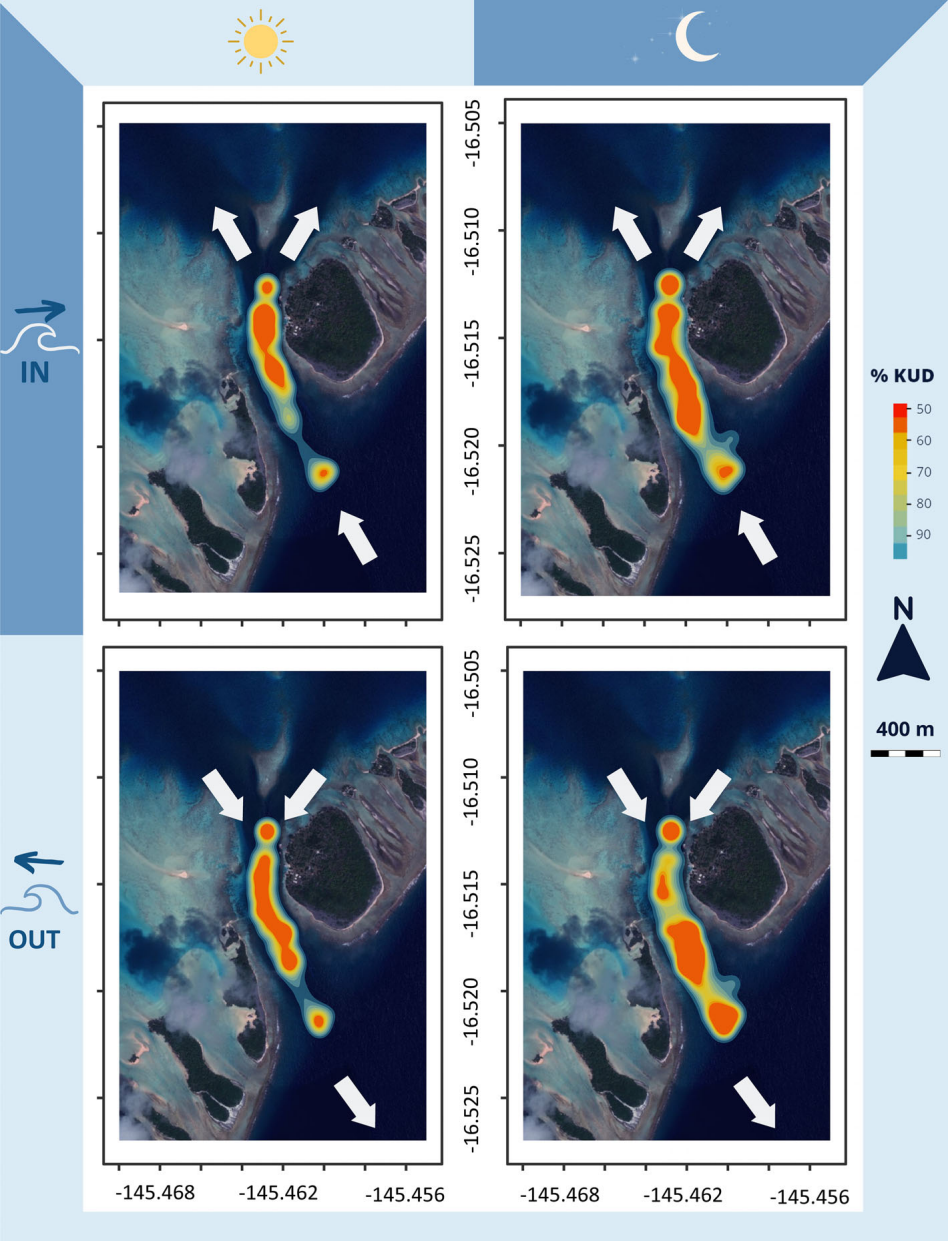
Changes in space use (kernel utilization density, KUD) over diel (day/night) and tidal (incoming/outgoing) cycles for 37 *C. amblyrhynchos* tagged in the South Pass of Fakarava (one shark had fewer than five positions and was removed from the analyses). White arrows highlight the direction of tidal currents.

For the core space use (50% KUD), the top‐ranked model (wAIC_c_ = 0.97) included diel and tidal cycles (Table 1a). Based on the models' output, sharks used larger areas during the night (0.036 ± 0.018 km^2^) than during the day (0.028 ± 0.012 km^2^; Figure 2a). Incoming currents also tended to increase the area used by sharks (0.033 ± 0.015 km^2^) compared to outgoing currents (0.030 ± 0.016 km^2^; Figure 2a), but the core areas were more variable and did not seem to differ across tidal states. However, the location of the core areas changed throughout the diel and tidal cycles. During the day, sharks were scattered throughout the channel during outgoing currents, but were concentrated in the northern region of the channel during incoming currents (Figure 3). The space use of sharks moved towards the southern part of the channel during the night.

**TABLE 1:** Summary of generalized linear mixed models estimating the influence of abiotic variables (diel and tidal cycles) on *Carcharhinus. amblyrhynchos* (a) 50% and (b) 95% kernel utilization density in the South Pass of Fakarava.

Model	*df*	logLik	AIC_c_	ΔAIC_c_	wAIC_c_	*R*_m_ (%)	*R*_c_ (%)
(a) 50% KUD_log_							
**~Diel + Tide + (1|ID) + (1|Month)**	**6**	**−702.8**	**1417.7**	**0.0**	**0.967**	**7.58**	**53.38**
~Diel × Tide + (1|ID) + (1|Month)	7	−705.2	1424.4	6.7	0.033	5.77	41.53
~Diel + (1|ID) + (1|Month)	5	−718.3	1446.6	28.9	0.000	4.36	40.15
~ Tide + (1|ID) + (1|Month)	5	−755.3	1520.6	102.9	0.000	1.41	37.04
~(1|ID) + (1|Month) *Null*	4	−769.6	1547.3	129.6	0.000	0.00	35.65
~(1|ID)	3	−781.5	1569.1	151.4	0.000	0.00	33.80
~(1|Month)	3	−1053.2	2112.4	694.7	0.000	0.00	1.51
(b) 95% KUD							
**~Diel × Tide + (1|ID) + (1|Month)**	**7**	**−18,925.0**	**37,864.0**	**0.0**	**1.000**	**7.58**	**53.38**
~Diel + Tide + (1|ID) + (1|Month)	6	−18,936.0	37,884.1	20.1	0.000	7.47	53.29
~Diel + (1|ID) + (1|Month)	5	−18,959.4	37,928.9	64.9	0.000	6.58	52.38
~Tide + (1|ID) + (1|Month)	5	−19,046.2	38,102.5	238.5	0.000	0.90	46.80
*Null (ID + Month)*	4	−19,067.9	38,143.9	279.9	0.000	0.00	45.90
*ID*	3	−19,086.9	38,179.8	315.8	0.000	0.00	43.78
*Month*	3	−19,479.0	38,963.9	1099.9	0.000	0.00	1.75

Abbreviations: ΔAIC_c_, difference in AIC_c_ between the current and top‐ranked model; Akaike's information criterion corrected for small sample size; *df*, degree of freedom; *R*_c_ conditional *R* ^2^ (fixed and random effects); *R*_m_, marginal *R* ^2^ (fixed effects); *wAIC*_c_, model probability.

Even though the total variance explained by the top‐ranked model was 53.4%, most of this was from the random factors (ID and months), with only 7.6% explained by the fixed factors. The diel cycle seemed to have more influence on space use (*R*_m_ = 4.4%) compared to tidal cycle (*R*_m_ = 1.4%), whereas individual variations appeared to explain more of the variability in area size (~33.8%) than months did (~1.5%; Table 1a).

For the space use (95% KUD), the top‐ranked model (wAIC_c_ = 1.00) included the interaction between the diel and tidal cycles (Table 1b). Tides affected space use more at nighttime, i.e. larger space use during incoming than outgoing currents, compared to daytime, i.e. similar space use across tides (Figure 2b). Similar to the core space, the diel cycle had greater influence on space use (*R*_m_ = 6.6%) than the tidal cycle (*R*_m_ = 0.9%). Most of the top‐ranked model was also explained by the random factors (~45.9%) where individual variation and months explained ~43.8% and ~1.8% of the variance, respectively (Table 1b).

Removing June and July from the data, did not change the outputs of the models (Figures S7 and S8, Table S2), suggesting that the grouper aggregation did not significantly modify the spatial behavior of sharks.

### 3.2 Activity pattern

The top‐ranked model (wAIC_c_ = 1.00) included the lunar cycle and the interaction between the tidal cycle and the diel cycle (Table 2a). The total variance explained by the top‐ranked model was 15.3%, whereas the total variance explained by the null model including the random factors (ID and months) was 5.7%. Shark ID had more influence on activity (*R*_c_ = 5.2%; Table 2) than months (*R*_c_ = 0.6%; Table 2a).

**TABLE 2:** Summary of generalized additive mixed models estimating the influence of abiotic variables (diel, tidal, and lunar cycles) on (a) *Carcharhinus amblyrhynchos* activity and (b) depth in the South Pass of Fakarava.

Model	*df*	AIC_c_	ΔAIC_c_	wAIC_c_	*R*_m_ (%)	*R*_c_ (%)
(a) Acceleration_log_						
**~s(Hour × Tide) + s(Moon)**	**69**	**150,777.4**	**0.0**	**1.0**	**9.6**	**15.3**
~s(Hour × Tide) + s(Moon × Tide)	74	150,895.1	117.7	0.0	9.5	15.2
~s(Hour) + s(Moon × Tide)	64	151,836.1	1058.7	0.0	9.1	14.8
~s(Hour) + s(Moon) + Tide	60	151,941.7	1164.2	0.0	9.1	14.8
~s(Hour × Tide)	58	152,818.4	2040.9	0.0	8.7	14.4
~s(Hour) + Tide	56	154,655.4	3878.0	0.0	7.9	13.6
~s(Moon × Tide)	58	155,089.0	4311.5	0.0	7.7	13.4
~s(Moon + Tide)	54	155,552.6	4775.2	0.0	7.5	13.2
~s(Hour)	56	166,825.2	16,047.8	0.0	2.3	8.0
~s(Moon)	56	168,823.3	18,045.8	0.0	1.3	7.0
*Null (ID + Month)*	49	171,579.5	20,802.0	0.0	0.0	5.7
*ID*	38	172,538.7	20,802.0	0.0	0.0	5.2
*Month*	13	181,677.5	30,900.1	0.0	0.0	0.6
(b) Depth						
**~s(Hour × Tide) + s(Moon × Tide)**	**78**	**758,665.6**	**0.0**	**1.0**	**14.6**	**37**
~s(Hour × Tide) + s(Moon)	67	758,899.6	233.9	0.0	14.6	37.5
~s(Hour × Tide)	65	758,905.2	239.6	0.0	14.5	37.4
~s(Hour) + s(Moon) + Tide	60	759,477.8	812.1	0.0	14.2	37.1
~s(Hour) + Tide	58	759,483.4	817.8	0.0	14.2	37.1
~s(Hour) + s(Moon × Tide)	68	760,332.9	1667.2	0.0	13.8	36.7
~s(Hour)	53	762,234.9	3569.3	0.0	12.9	35.8
~s(Moon × Tide)	65	779,724.2	21,058.6	0.0	3.5	26.4
~s(Moon) + Tide	53	783,222.5	24,556.9	0.0	1.4	24.3
~s(Moon)	52	785,660.3	26,994.7	0.0	0.0	22.9
*Null (ID + Month)*	50	785,688.8	27,023.1	0.0	0.0	22.9
*ID*	38	786,968.0	28,302.3	0.0	0.0	22.1
*Month*	14	818,030.6	59,364.9	0.0	0.0	0.7

*Note*: Italic indicates null models. Bold indicates the selected model.

Abbreviations: ΔAIC_C_, difference in AIC between the current and top‐ranked model; AIC, Akaike's information criterion corrected for small sample size; *df*, degree of freedom; *R*_c_, conditional *R* ^2^ (fixed and random effects); *R*_m_, marginal *R* ^2^ (fixed effects); wAIC_c_, model probability.

**FIGURE 4:**
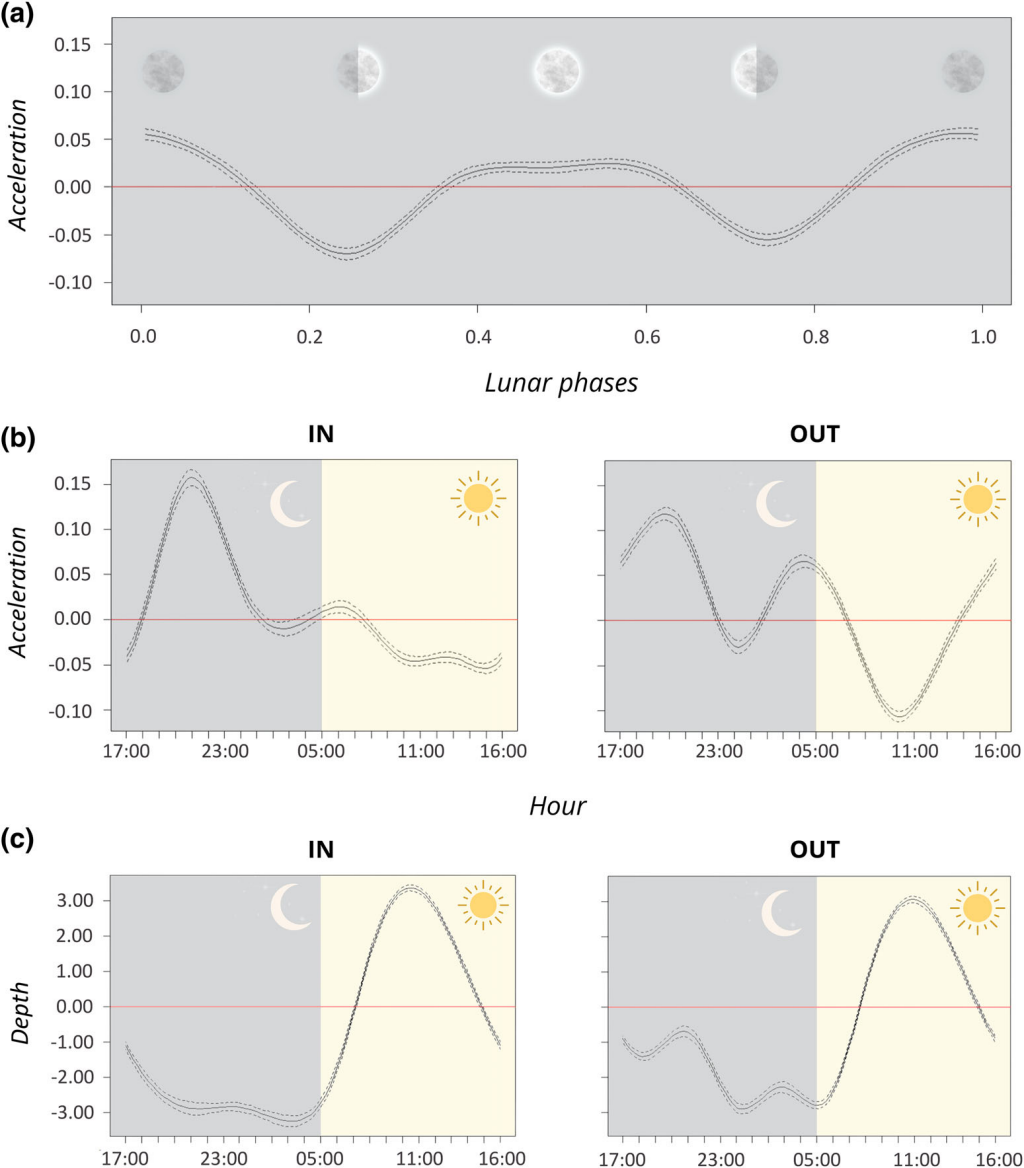
Spatio‐temporal dynamics of *C. amblyrhynchos* activity (acceleration) and depth. (a) Partial effect plot of acceleration model according to lunar phases. (b) Partial effects of time of day and tidal cycle on shark activity. (c) Partial effects of time of day and tidal cycle on shark depth.

The interaction between the diel and tidal cycles had the most influence on activity (*R*_c_ = 14.4%). Sharks were more active at night (mean ± standard deviation = 0.716 ± 0.405 m s^−2^) than during the day (0.644 ± 0.266 m s^−2^; Figure 4). Globally, activity was higher during outgoing currents in the daytime and nighttime (0.711 ± 0.286 m s^−2^ and 0.780 ± 0.432 m s^−2^, respectively) compared to the incoming currents (0.572 ± 0.222 m s^−2^ and 0.646 ± 0.360 m s^−2^, respectively; Figure S9). The effect of tides was stronger during the day, with activity slowly decreasing through the day during incoming current, but with strong peaks and troughs during outgoing currents (GAMM; *F*_IN,37,6_ = 932.9, *p*_IN_ < 0.001, *F*_OUT,37,6_ = 924.8, *p*_OUT_ < 0.001; Table 2a and Figure 4b). Sharks were also most active during the full and new moons (GAMM; *F*_37,7_ = 307.1, *p* < 0.001; Figure 4a).

**FIGURE 5:**
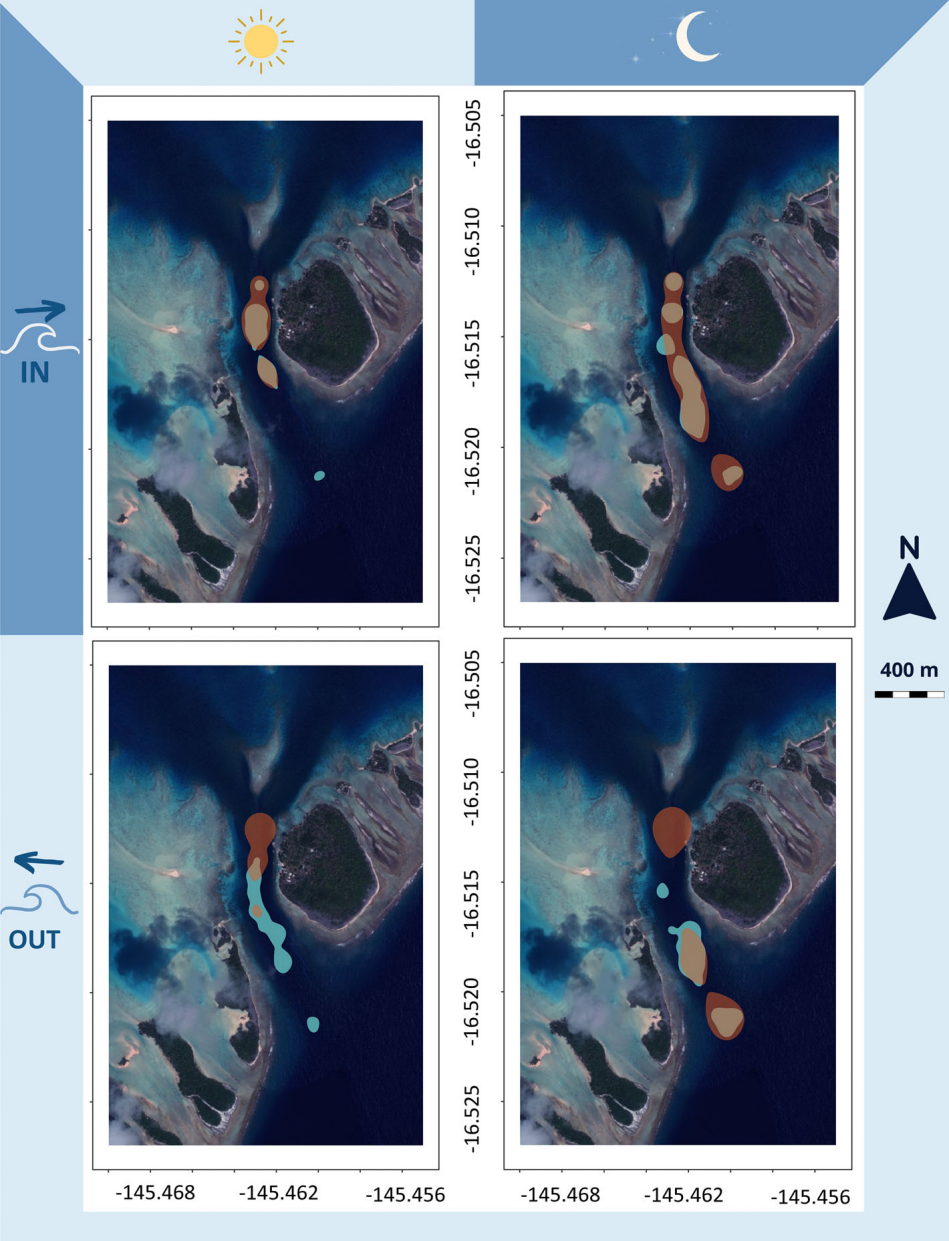
Spatial distribution of regions with high activity values in orange and low activity values in blue. Upper panels show incoming current and lower panels show outgoing current. Left column shows daytime and right column shows nighttime.

Regions of high and low activity varied with the diel cycle. During the night, regions of high and low activity were homogeneously distributed throughout the channel during the incoming current and mostly focused within the southern entrance of the channel during outgoing current. During the day, regions of high and low activity overlapped towards the northern end of the channel during incoming current, but regions of low activity moved away from the lagoon side during outgoing current. Additionally, high activity was not observed at the southern entrance of the channel during daytime (Figure 5).

Removing June and July from the data, did not change the outputs of the models (Figures S10 and S11, Table S3), suggesting that the grouper aggregation did not significantly modify the patterns of acceleration in sharks.

### 3.3 Depth pattern

The top‐ranked model (wAIC_c_ = 1.00) included the interaction between the tidal cycle and both the diel and lunar cycles (Table 2b). The total variance explained by the top‐ranked model was 37.5%, whereas the total variance explained by the null model including the random factors (ID and months) was 22.9%. Shark ID had more influence on depth (*R*_c_ = 22.1%; Table 2b) than months (*R*_c_ = 0.7%; Table 2b).

The interaction between diel and tidal cycle had the second largest influence on swimming depth (*R*_c_ = 37.4%; Table 2b). Sharks were deeper during the day (18.56 ± 5.94 m) than at night (16.09 ± 5.38 m) and were deepest around 11:00 AM. Although swimming depth was significantly affected by tides (GAMM; *F*_IN,37,7_ = 17,617.1, *p*_IN_ < 0.001, *F*_OUT,37,7_ = 9423.9, p_OUT_ < 0.001), there was only a small depth difference during the day between outgoing and incoming currents (17.65 ± 5.94 and 19.59 ± 5.78 m, respectively) and at night (16.04 ± 5.79 and 16.13 ± 4.90 m, respectively; Figure S12). Lunar phase and tides also significantly affected swimming depth (GAMM; *F*_IN,37,7_ = 20.5, *p*_IN_ < 0.001, *F*_OUT,37,7_ = 34.9, *p*_OUT_ < 0.001), but the deviance explained was not improved when the lunar phase was included in the model (Table 2b, Figures S12 and S13).

Removing June and July from the data, did not change the outputs of the models (Figure S14 and Table S3), suggesting that the grouper aggregation did not significantly modify the patterns of depth use in sharks.

## 4 DISCUSSION

This study provides novel insights into the space use, activity patterns, and swimming depth of a large aggregation of *C. amblyrhynchos*. The south channel of the Fakarava atoll is a dynamic environment, where *C. amblyrhynchos* can both forage and find an energy refuge. We show that shark behavior varies spatiotemporally over diel, tidal, and lunar cycles and highlight that this may create spatial heterogeneity in predation pressure. Our initial hypotheses were verified as our findings demonstrated that (1) space use and activity are higher at night regardless of the tide regime, suggesting nocturnal foraging activity, (2) activity is also higher during outgoing currents regardless of the time of the day due to turbulence, and (3) activity increases during full and new moons. Swimming depths were shallower during the day, but tide only had a slight effect on shark depth.

The combined effect of diel and tidal cycles was the strongest driver of the spatio‐temporal habitat use and activity dynamics of *C. amblyrhynchos*. During the day, sharks remain in restricted areas while at night they were more active and used a larger proportion of the area available in the channel, probably to forage (Labourgade et al., 2020; Mourier et al., 2016; Papastamatiou et al., 2021). These animals are obligate ram ventilators (except in rare cases; see Bullock et al., 2023), negatively buoyant, and must swim continuously to obtain enough oxygen to meet metabolic demands. Swimming in updraft currents created by tides offers them a strategy to reduce their swimming effort and subsequent energetic costs during the day (Papastamatiou et al., 2021). Up to four updraft zones may form during incoming tides. During outgoing tides, only one updraft zone persists at the center of the channel and the flow becomes highly turbulent (Papastamatiou et al., 2021, see Figure S3). The number of optimal updrafts varies over time depending on current strength (i.e., they are not used by sharks if the strength of the updraft is too low), which is influenced by swell conditions (strength and direction) in Fakarava. As a result, the individuals are further apart when using turbulent zones during outgoing tides, but the space use area of each individual tends to be more restricted (50% KUD) than during incoming tides. Activity was also higher during outgoing tides due to turbulence causing the sharks to bounce around, although muscular activity appears minimal (for more details, see Papastamatiou et al., 2021).

Similar to other locations, *C. amblyrhynchos* display nocturnal peaks in activity (McKibben & Nelson, 1986; Papastamatiou et al., 2018). At night during incoming tides, the peak of activity occurred at twilight which differed from *C. amblyrhynchos* in Palmyra atoll, where activity peaked in the middle of the night (Papastamatiou et al., 2018). High‐activity phases were spread over the entire channel compared to the day, which suggests hunting activity across the channel. In addition to costs incurred from foraging activity, turbulence created by outgoing currents appears to affect sharks' movement costs, decisions, and behaviors, explaining the different patterns observed in our study. Due to turbulence during outgoing tides, the energetic cost of foraging for sharks should increase in the shallower north part of the channel, where turbulence will be stronger within downdraft zones (Papastamatiou et al., 2021). This in turn may influence prey behavior as the foraging cost of predation in the northern region should decrease during outgoing tides (Papastamatiou et al., 2023). We thus suggest that sharks have to manage the cost induced by turbulence by shifting their foraging activity to the south part of the channel where turbulence is less pronounced (Higham et al., 2015). As such, the shallower northern region of the channel may be safer at night for reef fishes, especially during outgoing tides. High‐activity phases were found to split into three distinct areas at night and during outgoing tides, where two of them are new compared to daytime. One is located in the updraft current zone and one at the entrance of the channel near the drop off, where the depth can reach 50 m (see Figures S2 and S3). Thus, it is possible that sharks hunt in the deeper zones near the drop off where turbulence is weaker before resting on the upward current zone between hunting sessions. The depth data are difficult to compare temporally and spatially to acceleration data as they are measured at a lower rate. However, sharks seem to use shallower waters at night, which could reflect either foraging on fish higher in the water column or more frequent changes in depth at night (due to chasing prey vertically; Labourgade et al., 2020).

In Fakarava, we observed increased activity during the full and new moons, which is likely driven by the lunar‐induced stronger tidal currents (Papastamatiou et al., 2015). These moon phases are also periods when several reef fishes can form spawning aggregations (Bijoux et al., 2013; Mourier et al., 2019; Weideli et al., 2015; Wilson et al., 2010), providing important foraging opportunities. Fish spawning aggregations can also influence the habitat selection of predators (Pickard et al., 2016; Rhodes et al., 2019; Weideli et al., 2015), and grouper spawning aggregations, which only occur during summer months, seem to represent a significant energy source for *C. amblyrhynchos* (Mourier et al., 2016). However, these aggregations do not alter the behaviour and activity of *C. amblyrhynchos* within the pass (Figure S7, S8, S10, S11, and S14, Tables S2 and S3). Other reef fish species also form fish‐spawning aggregations year‐round (e.g. Convict surgeon fish [*Acanthurus triostegus*, Linnaeus 1758] spawning on new and full moons). These fish‐spawning aggregations could be preyed on by *C. amblyrhynchos* and could supplement the subsidies they receive from grouper entering the channel from the lagoons (Mourier et al., 2016).

Individual variability was evident, with shark ID being the main factor influencing space use, activity, and depth. This supports previous studies showing that intraspecific behavioral variation in sharks is common, including evidence of individual specialization in diet, time of peak activity, and horizontal and vertical habitat use (Baremore et al., 2021; Matich et al., 2011; Papastamatiou, Leos‐Barajas, et al., 2022a). For instance, sharks may display behavioral plasticity with individuals within a population showing a wide range of individual activity patterns (Papastamatou, Mourier, et al., 2022b). Differences between individuals could also be due to internal factors such as sex, age (although unlikely in our study as all sharks were mature and 34 of 38 were females; Tables S1, S4, and S5) or hunger (a shark catching a large prey one night does not necessarily need to hunt on the next night as its energy requirements may be met). Sharks may also form unique sociospatial groups with different strategies within the channel (Papastamatiou et al., 2020). Thus, future work will be necessary to study individual and social behaviors to investigate behavioral variations at the individual level.

We found that times and locations of increased activity in reef sharks in the channel can at least be partially explained by diel period and tidal states. *C. amblyrhynchos* foraging behavior is dynamic, with sharks hunting almost exclusively at night but also being regulated by tidal states. If increased activity is the result of the combined effects of turbulence produced by outgoing currents and foraging activity, our study provides quantitative information on how the energy landscape modulates the spatial distribution of hunting in a marine predator. A recent paradigm highlighted how dynamic energy landscapes of predators may modulate the fearscape of their prey (Papastamatiou et al., 2023). Although we did not measure prey fearscapes, we do show how the location of shark hunting activity (and likely risk) will vary with the energy landscape of predators through the tidal cycle (e.g., there may be little hunting in the northern region of the channel during outgoing tides). The interaction between the energy and fear landscapes may create spatiotemporal heterogeneity in the predatory role of sharks, which may cascade through food webs.

## AUTHOR CONTRIBUTIONS

J.M., Y.P.P., C.H., L.B., and S.P. conceived the study. A.L. and J.M. analyzed the data. Y.P.P., C.H., L.B., and J.M. performed the fieldwork. A.L. and J.M. wrote the paper with input from all authors.

## FUNDING INFORMATION

Funding was provided by the Blancpain Ocean Commitment. Sponsors supporting the expedition include Tahiti Tourisme, Air Tahiti Nui, Aqualung, Nikon, Seacam, Aqualung, and ApDiving.

## Supporting information


**Data S1:** Supporting information.
