# Supplementary material for: Abiotic drivers of the space use and activity of gray reef sharks Carcharhinus amblyrhynchos in a dynamic tidal environment
Source: J Fish Biol. 2024 May 29;106(5):1520–30. doi: 10.1111/jfb.15825 (PMC12120322; doi:10.1111/jfb.15825)
Supplement: Supplementary file 1 — Data S1: Supporting information. [file JFB-106-1520-s001.docx]

**Supplementary material**

**Abiotic drivers of space use and activity of grey reef sharks *Carcharhinus amblyrhynchos* in a dynamic tidal environment**

Anaïs Laurioux^1^, Charlie Huveneers^2^, Yannis Papastamatiou^3^, Serge Planes^4^, Laurent Ballesta^5^, Johann Mourier^1^

^1^ MARBEC, Univ Montpellier, CNRS, IFREMER, IRD, Sète, France

^2^College of Science and Engineering, Flinders University, Bedford Park, South Australia, Australia

^3^Institute of the Environment, Department of Biological Sciences, Florida International University, North Miami, FL, USA

^4^PSL Research University, EPHE-UPVD-CNRS, UAR 3278 CRIOBE, Université de Perpignan, Perpignan Cedex, France

^5^Andromède Océanologie, Carnon, France


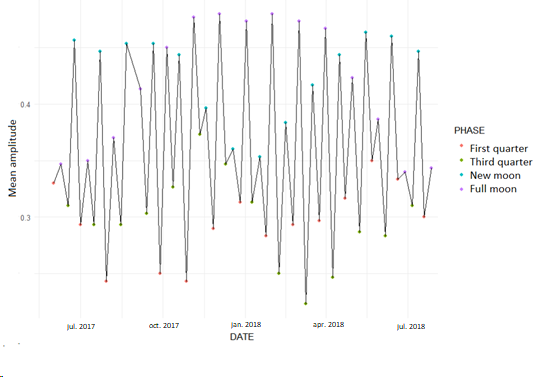


Figure S1: Mean amplitude of sea-level according to lunar phases in meters during the study period.

*
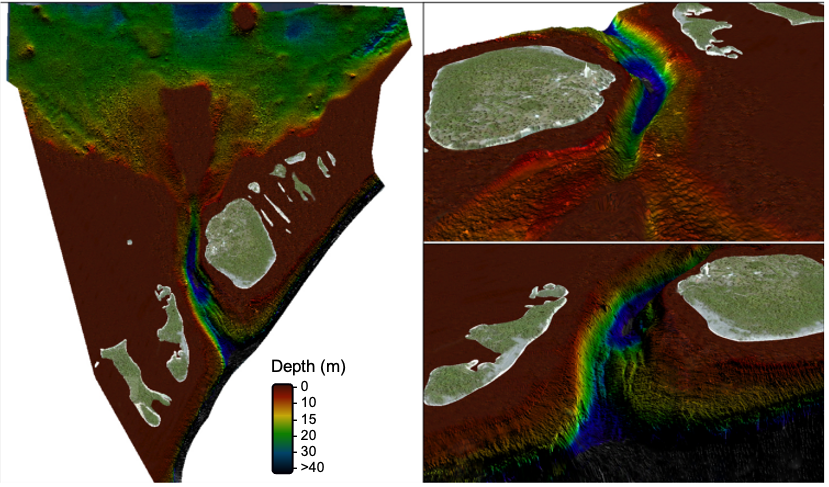
*


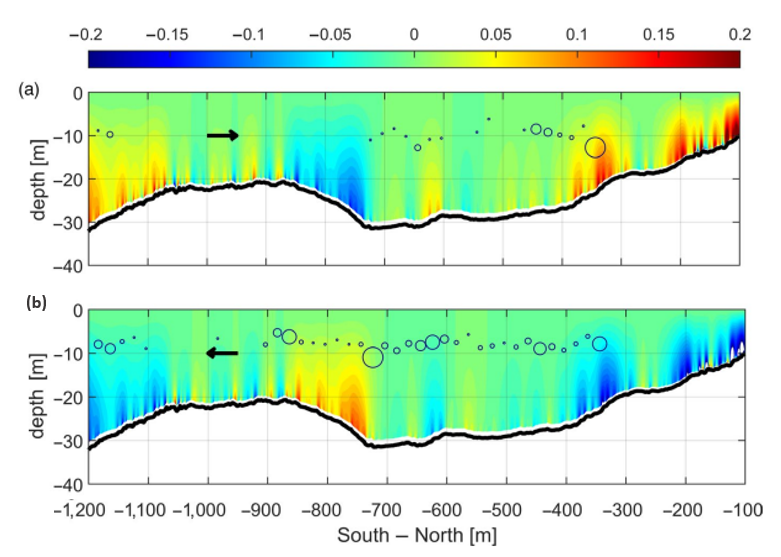
Figure S2: 3D Bathymetry map. Bathymetric measurements of the channel using a GeoSwath multibeam sonar system (MBSS).

Figure S3: Updraft currents predicted in the pass of Fakarava from a figure in Papastamatiou et al, 2021. The gradient indicates locations of updraft currents during (a) incoming tides and (b) outgoing tides. Color contours are the predicted ratio of vertical and tidal velocities.


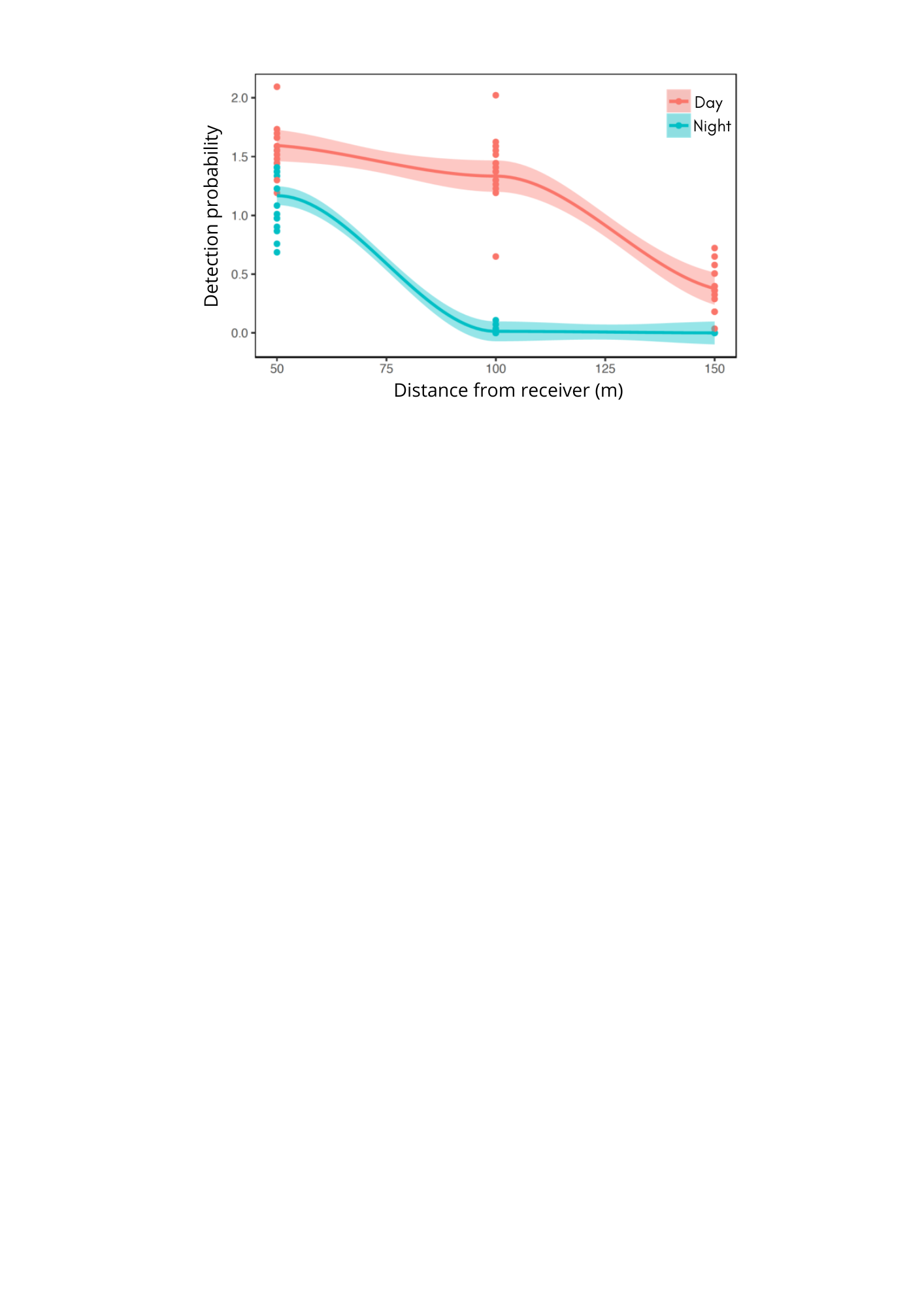
Figure S4: Detection range of receivers according to diel cycle and tag’s distance from receivers.


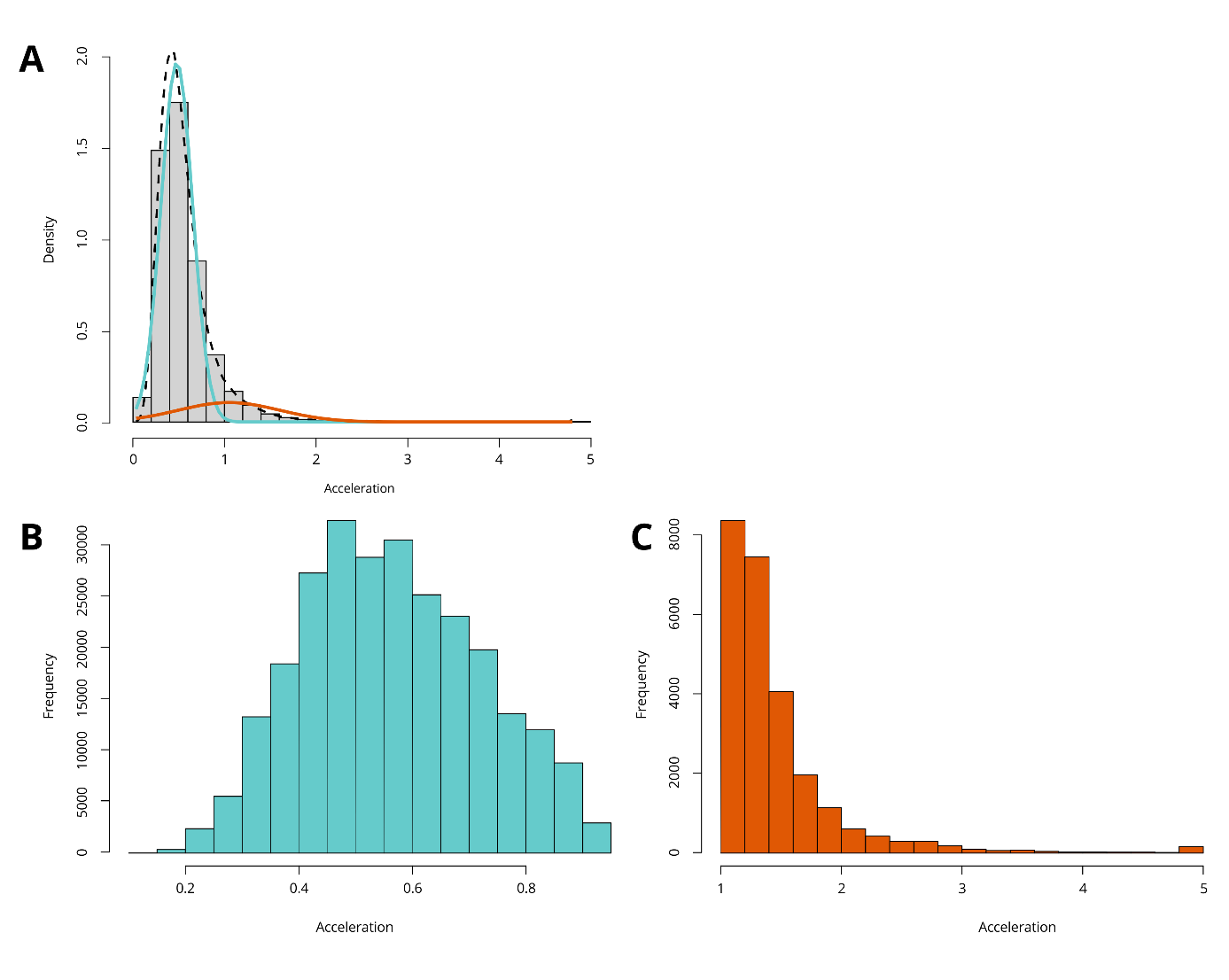


Figure S5: (A) The frequency distribution of acceleration values for all sharks. Most acceleration were weak (blue) but there were a small number of strong acceleration values (orange). Frequency distribution of acceleration value with posterior probability greater than 0.75 to be associated with low (B) and high values (C).

*
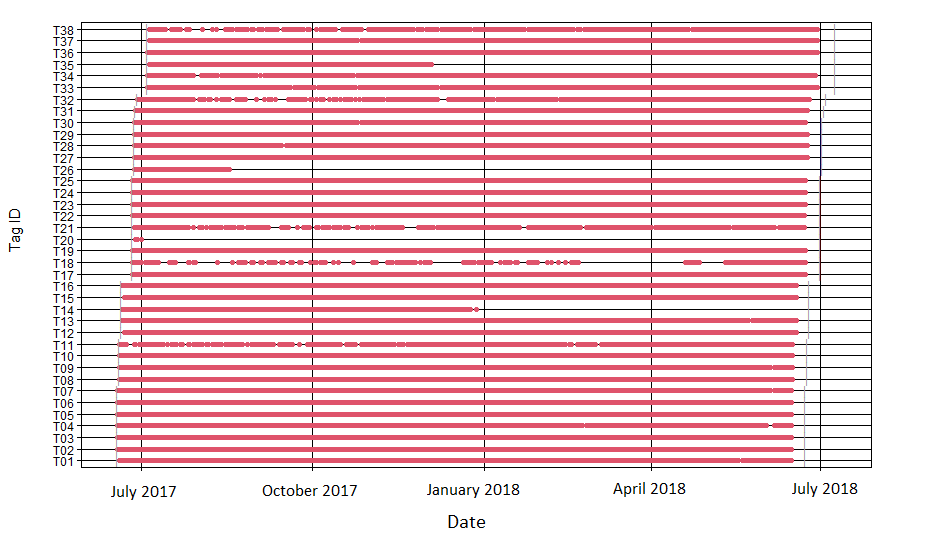
*Figure S6: Abacus plot of detections of the 38 grey reef sharks in the network of receivers in the pass.

Table S1: Tagging-sharks metadata across our study period. T20 has been removed from our analysis, as the number of detections was too low. This shark aggregation is characterized by a female-biased sex ratio (F:M = 4:1; Mourier et *al*., 2016), explaining the greater number of females tagged.

| **ID** | **Sex** | **Total length (cm)** | **Days detected** | **Number of detections** | **Residency index** |
| --- | --- | --- | --- | --- | --- |
| T01 | F | 145 | 349 | 61784 | 0.96 |
| T02 | F | 147 | 349 | 46981 | 0.96 |
| T03 | F | 136 | 350 | 59555 | 0.96 |
| T04 | F | 161 | 346 | 87811 | 0.95 |
| T05 | F | 129 | 350 | 89958 | 0.96 |
| T06 | F | 137 | 350 | 94751 | 0.96 |
| T07 | F | 140 | 349 | 59023 | 0.96 |
| T08 | F | 138 | 351 | 28650 | 0.96 |
| T09 | F | 150 | 350 | 37497 | 0.96 |
| T10 | M | 137 | 351 | 54386 | 0.96 |
| T11 | F | 137 | 317 | 12264 | 0.87 |
| T12 | F | 140 | 353 | 90041 | 0.97 |
| T13 | F | 152 | 352 | 30535 | 0.97 |
| T14 | F | 143 | 179 | 18015 | 0.49 |
| T15 | F | 115 | 353 | 51809 | 0.97 |
| T16 | F | 136 | 353 | 151716 | 0.97 |
| T17 | F | 133 | 358 | 26907 | 0.98 |
| T18 | F | 148 | 160 | 23186 | 0.44 |
| T19 | F | 140 | 358 | 59989 | 0.98 |
| T20 | F | 165 | 1 | 1 | 0.00 |
| T21 | F | 142 | 297 | 22770 | 0.82 |
| T22 | F | 140 | 357 | 54423 | 0.98 |
| T23 | F | 139 | 358 | 137743 | 0.98 |
| T24 | F | 125 | 358 | 43704 | 0.98 |
| T25 | F | 128 | 358 | 63815 | 0.98 |
| T26 | F | 147 | 48 | 19260 | 0.13 |
| T27 | F | 140 | 359 | 124216 | 0.99 |
| T28 | F | 150 | 357 | 60036 | 0.98 |
| T29 | F | 143 | 359 | 69232 | 0.99 |
| T30 | F | 154 | 357 | 25976 | 0.98 |
| T31 | F | 132 | 359 | 40083 | 0.99 |
| T32 | M | 143 | 310 | 7000 | 0.85 |
| T33 | F | 161 | 357 | 16626 | 0.98 |
| T34 | M | 143 | 351 | 17532 | 0.96 |
| T35 | F | 135 | 153 | 7134 | 0.42 |
| T36 | M | 132 | 360 | 79031 | 0.99 |
| T37 | F | 133 | 359 | 26910 | 0.99 |
| T38 | F | 145 | 307 | 12318 | 0.84 |

Table S2: Summary of generalized linear mixed models estimating the influence of abiotic variables (diel and tidal cycles) on a) 50% KUDs and b) 95% KUDs without June and July. df: degree of freedom; AIC: Akaike’s information criterion corrected for small sample size; ΔAIC_C_: difference in AIC between the current and top-ranked model; *w*AIC_c_: model probability; R_m_: marginal R² (fixed effects); R_c_: conditional R² (fixed and random effects). As a principle of parsimony, the chosen model for 50% KUD took into account both the variance explained by the model and its complexity. Although the second model has slightly higher R²m and R²c, the increase in variance is not significant enough to retain this model. No differences were found for 95% KUD models, except the increase of individual’s variability.

| Model | df | logLik | AIC_c_ | ΔAIC_c_ | wAIC_c_ | R_m (%)_ | R_c_  _(%)_ |
| --- | --- | --- | --- | --- | --- | --- | --- |
| a) 50% KUD_log_ |  |  |  |  |  |  |  |
| ~ Diel + Tide + (1\|ID) + (1\|Month) | 6 | -548.6 | 1109.2 | 0.0 | 0.938 | 4.65 | 44.98 |
| ~ Diel x Tide + (1\|ID) + (1\|Month) | 7 | -550.3 | 1114.6 | 5.4 | 0.062 | 4.69 | 45.02 |
| ~ Diel + (1\|ID) + (1\|Month) | 5 | -559.0 | 1128.1 | 18.9 | 0.000 | 3.48 | 43.80 |
| ~ Tide + (1\|ID) + (1\|Month) | 5 | -584.6 | 1179.1 | 69.9 | 0.000 | 1.15 | 41.37 |
| ~ (1\|ID) + (1\|Month) | 4 | -594.1 | 1196.2 | 87.0 | 0.000 | 0.00 | 40.22 |
| ~ (1\|ID) | 3 | -597.4 | 1200.9 | 91.7 | 0.000 | 0.00 | 39.35 |
| ~ (1\|Month) | 3 | -873.7 | 1753.3 | 644.1 | 0.000 | 0.00 | 0.47 |
|  |  |  |  |  |  |  |  |
| 1. 95% KUD |  |  |  |  |  |  |  |
| ~ Diel x Tide + (1\|ID) + (1\|Month) | 7 | -15540.7 | 31095.4 | 0.0 | 1.000 | 5.84 | 57.34 |
| ~ Diel + Tide + (1\|ID) + (1\|Month) | 6 | -15550.6 | 31113.3 | 17.9 | 0.000 | 5.80 | 57.31 |
| ~ Diel + (1\|ID) + (1\|Month) | 5 | -15572.0 | 31154.0 | 58.6 | 0.000 | 4.94 | 56.42 |
| ~ Tide + (1\|ID) + (1\|Month) | 5 | -15628.7 | 31267.4 | 171.9 | 0.000 | 0.83 | 52.47 |
| ~ (1\|ID) + (1\|Month) | 4 | -15648.6 | 31305.1 | 209.7 | 0.000 | 0.00 | 51.61 |
| ~ (1\|ID) | 3 | -15657.6 | 31321.1 | 225.7 | 0.000 | 0.00 | 50.19 |
| ~ (1\|Month) | 3 | -16055.0 | 32116.1 | 1020.6 | 0.000 | 0.00 | 0.78 |


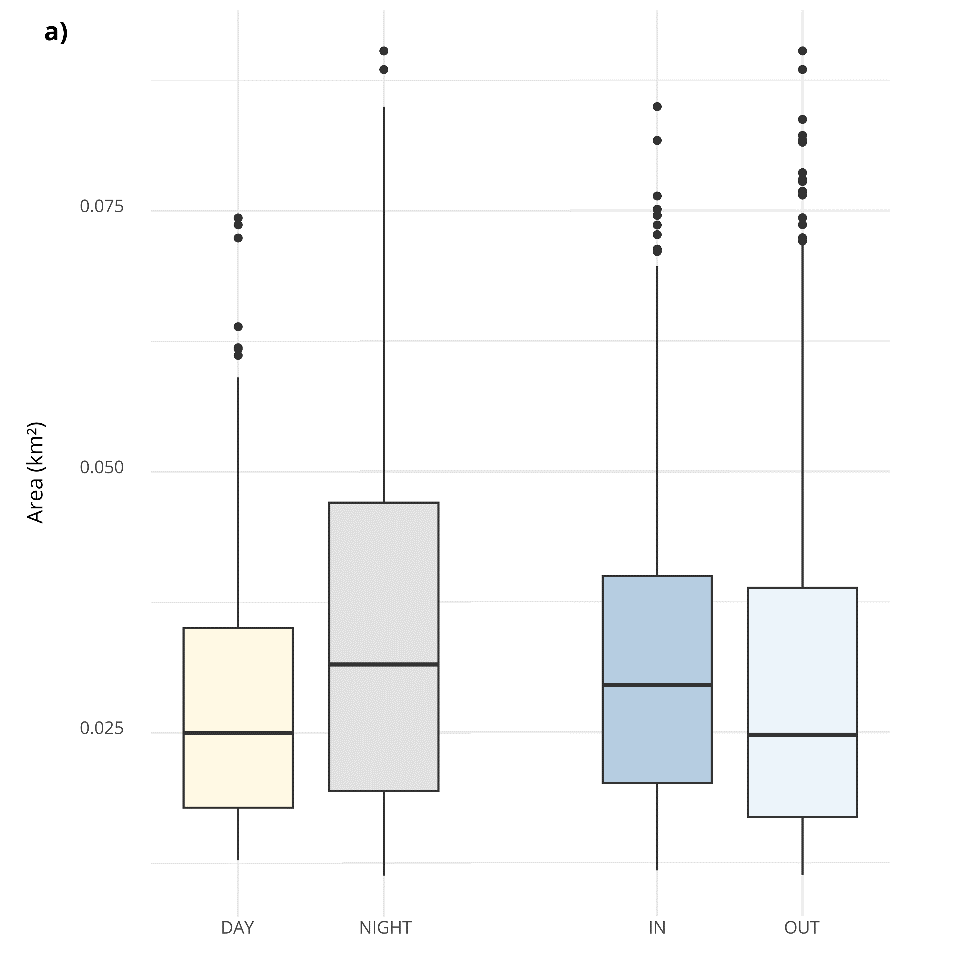


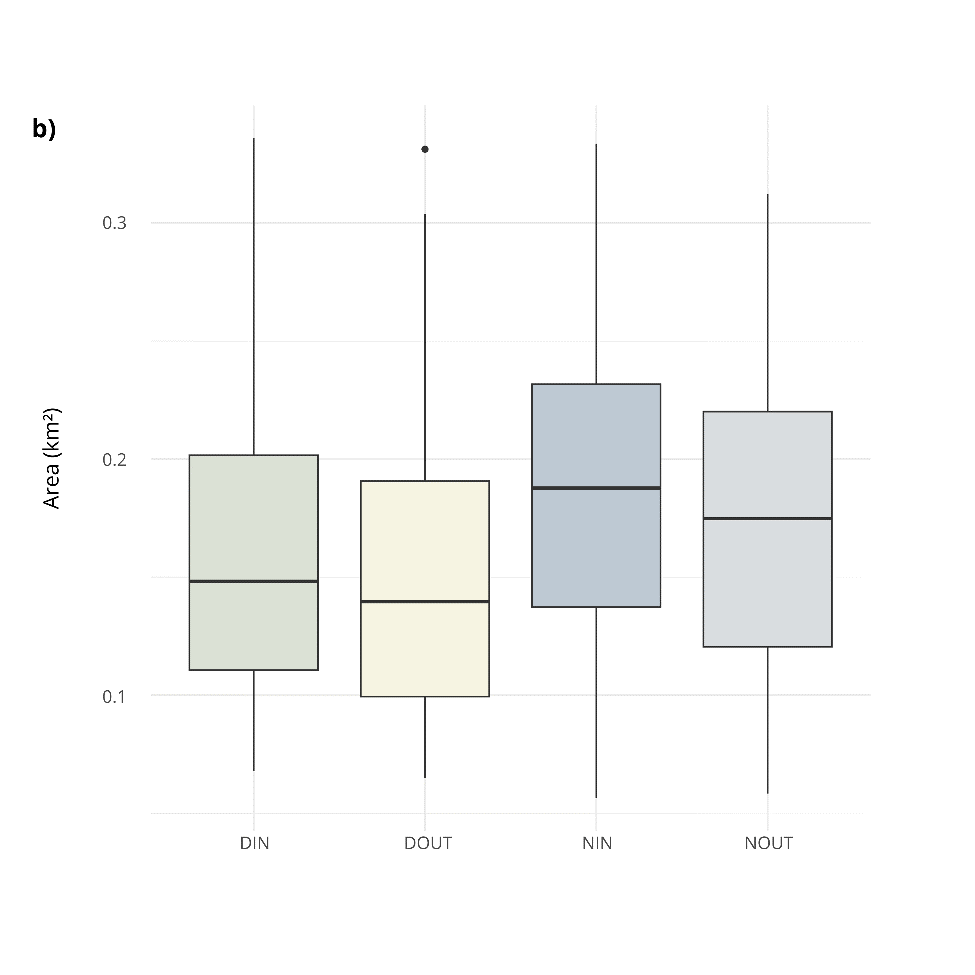


Figure S7: Effect of diel and tidal cycles effect on the size of a) 50% and b) 95% Kernel Utilization Density areas by grey reef sharks without June and July. DIN: incoming current at daytime, DOUT: outgoing current at daytime, NIN: incoming current at nighttime, NOUT: outgoing current at nighttime.


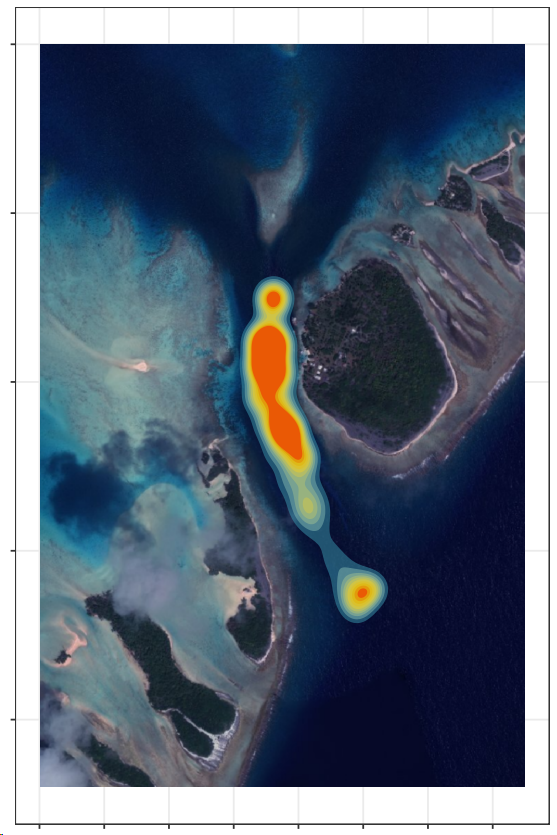
**DIN DOUT**


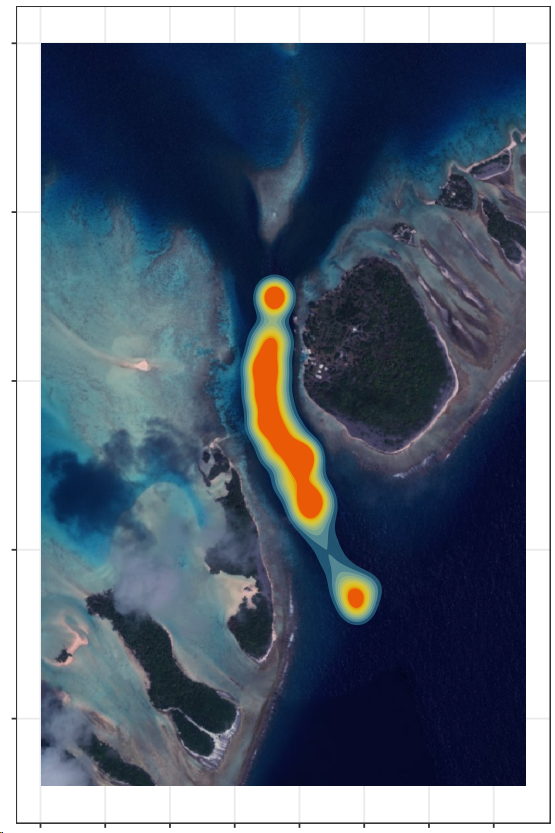


**NIN NOUT**


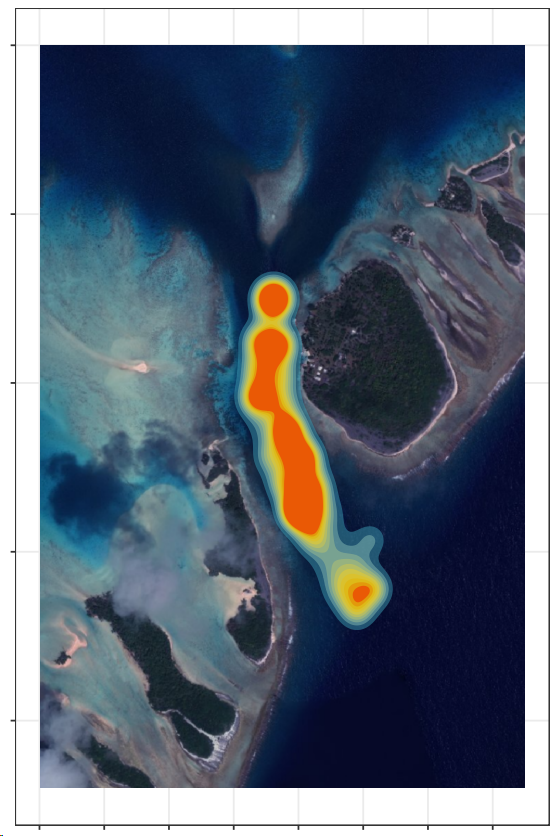

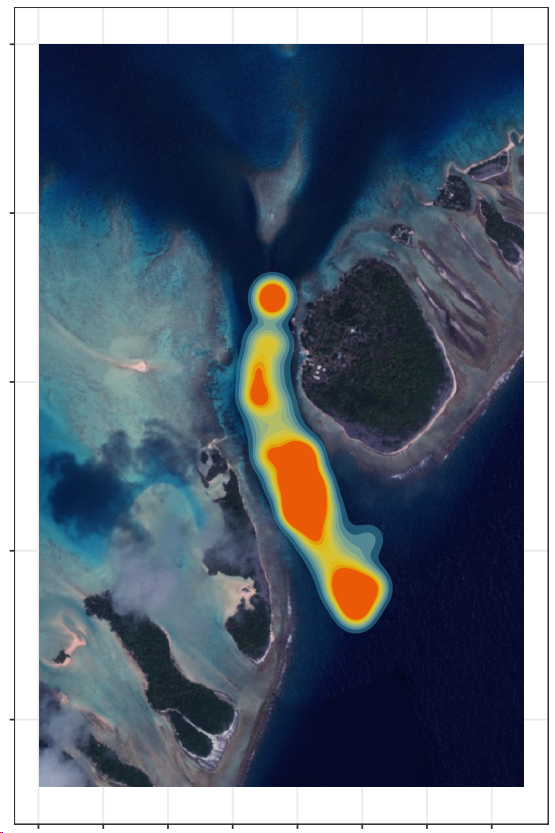


Figure S8: Changes in space use (KUD) over diel (day/night) and tidal (incoming/outgoing) cycles for 37 *Carcharhinus amblyrhynchos* tagged in the South Pass of Fakarava, without June and July (one shark have been removed from analyses as the number of detections was too low). White arrows highlight the direction of the current. DIN: incoming current at daytime, DOUT: outgoing current at daytime, NIN: incoming current at nighttime, NOUT: outgoing current at nighttime. Please see Figure in main manuscript for coordinates


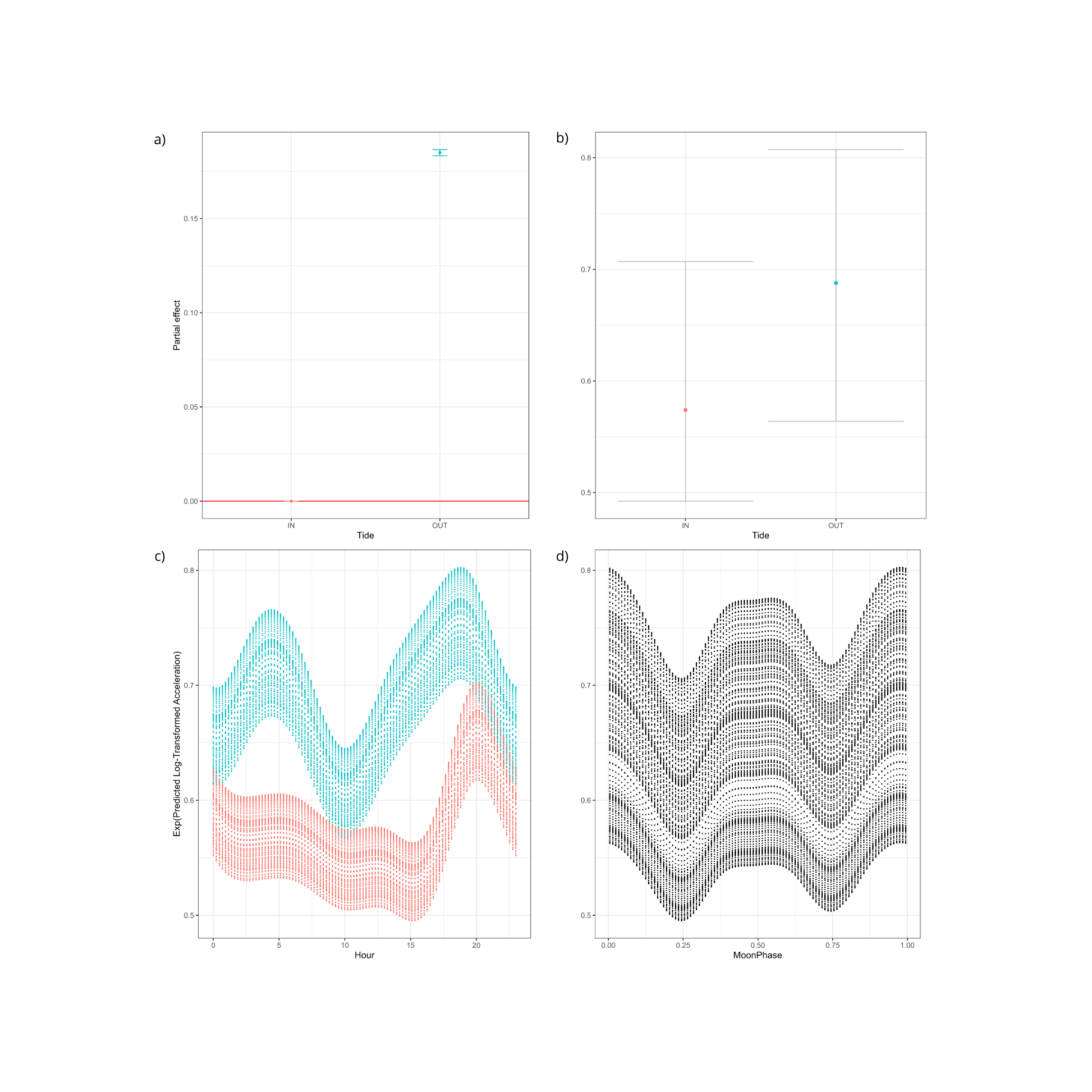


Figure S9: Acceleration model results. a) The influence of tides on acceleration; b) The mean of all predicted acceleration values and the minimum and maximum standard error among all predicted values; c) Predicted acceleration values according to hours and tides in the response scale; d) Predicted acceleration values according to moon phases and tides in the response scale.

Table S3: Summary of generalized additive mixed models estimating the influence of abiotic variables (diel, tidal, and lunar cycles) on a) grey reef sharks (*Carcharhinus amblyrhynchos*) activity and b) depth in the South Pass of Fakarava without June and July. df: degree of freedom; AIC: Akaike’s information criterion corrected for small sample size; ΔAIC_C_: difference in AIC between the current and top-ranked model; *w*AIC_c_: model probability; R_m_: marginal R² (fixed effects); R_c_: conditional R² (fixed and random effects). There are not many changes after removing June and July from our dataset. Same top models were selected for depth and acceleration.

| Model | df | AIC | ΔAIC | wAIC_c_ | Rm  _(%)_ | Rc  _(%)_ |
| --- | --- | --- | --- | --- | --- | --- |
| 1. Acceleration |  |  |  |  |  |  |
| ~ s(Hour x Tide) + s(Moon) | **65** | **125457.4** | **0.0** | **1.0** | **10.0** | **16.0** |
| ~ s(Hour x Tide) + s(Moon x Tide) | 64 | 125701.7 | 244.3 | 0.0 | 9.9 | 15.9 |
| ~ s(Hour) + s(Moon x Tide) | 59 | 126794.2 | 1336.8 | 0.0 | 9.3 | 15.3 |
| ~ s(Hour x Tide) | 58 | 126936.3 | 1478.9 | 0.0 | 9.3 | 15.3 |
| ~ s(Hour) + s(Moon) + Tide | 55 | 126962.5 | 1505.1 | 0.0 | 9.2 | 15.2 |
| ~ s(Hour) + Tide | 51 | 129591.5 | 4134.1 | 0.0 | 7.9 | 13.9 |
| ~ s(Moon x Tide) | 58 | 129662.8 | 4205.4 | 0.0 | 7.9 | 13.9 |
| ~ s(Moon) + Tide | 55 | 129907.6 | 4450.2 | 0.0 | 7.7 | 13.7 |
| ~ s(Hour) | 53 | 139404.5 | 13947.1 | 0.0 | 6.6 | 8.6 |
| ~ s(Moon) | 51 | 141646.4 | 16189.0 | 0.0 | 1.4 | 7.4 |
| *Null (ID + Month)* | 47 | 144186.9 | 18729.5 | 0.0 | 0.0 | 6.0 |

| *ID* | 38 | 144847.2 | 19389.7 | 0.0 | 0.0 | 5.6 |
| --- | --- | --- | --- | --- | --- | --- |
| *Month* | 11 | 153543.9 | 28086.5 | 0.0 | 0.0 | 0.5 |
|  |  |  |  |  |  |  |
| b) Depth |  |  |  |  |  |  |
| ~ s(Hour x Tide) + s(Moon x Tide) | **71** | **637495.2** | **0.0** | **1.0** | **14.6** | **39.0** |
| ~ s(Hour x Tide) + s(Moon) | 58 | 638211.8 | 716.6 | 0.0 | 14.6 | 38.6 |
| ~ s(Hour x Tide) | 56 | 638270.2 | 775.1 | 0.0 | 14.5 | 38.6 |
| ~ s(Hour) + s(Moon) + Tide | 55 | 638500.1 | 1004.9 | 0.0 | 14.2 | 38.5 |
| ~ s(Hour) + Tide | 53 | 638561.5 | 1066.3 | 0.0 | 14.2 | 38.4 |
| ~ s(Hour) + s(Moon x Tide) | 57 | 638635.0 | 1139.9 | 0.0 | 13.8 | 38.4 |
| ~ s(Hour) | 55 | 639498.5 | 2003.3 | 0.0 | 12.9 | 37.9 |
| ~ s(Moon x Tide) | 60 | 656458.1 | 18962.9 | 0.0 | 3.5 | 27.3 |
| ~ s(Moon) + Tide | 50 | 659534.0 | 22038.8 | 0.0 | 1.4 | 25.2 |
| ~ s(Moon) | 53 | 661472.6 | 23977.4 | 0.0 | 0.0 | 23.9 |
| *Null (ID + Month)* | 47 | 785688.8 | 24140.4 | 0.0 | 0.0 | 23.8 |
| *ID* | 38 | 786968.0 | 24986.8 | 0.0 | 0.0 | 23.2 |
| *Month* | 11 | 818030.6 | 52897.8 | 0.0 | 0.0 | 0.5 |


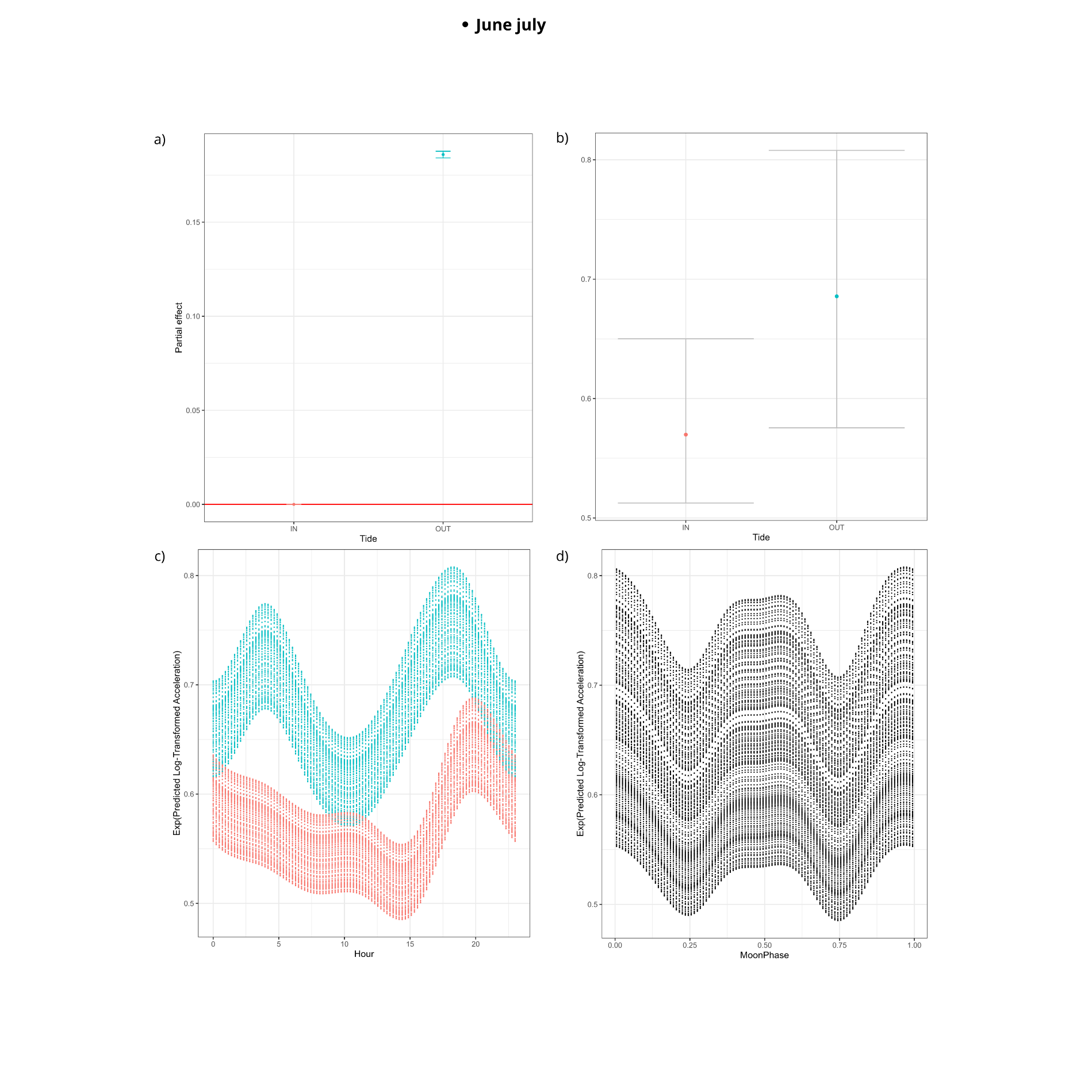


Figure S10: Acceleration model results without June and July. a) The influence of tides on acceleration; b) The mean of all predicted acceleration values and the minimum and maximum standard error among all predicted values; c) Predicted acceleration values according to hours and tides in the response scale; d) Predicted acceleration values according to moon phases and tides in the response scale.


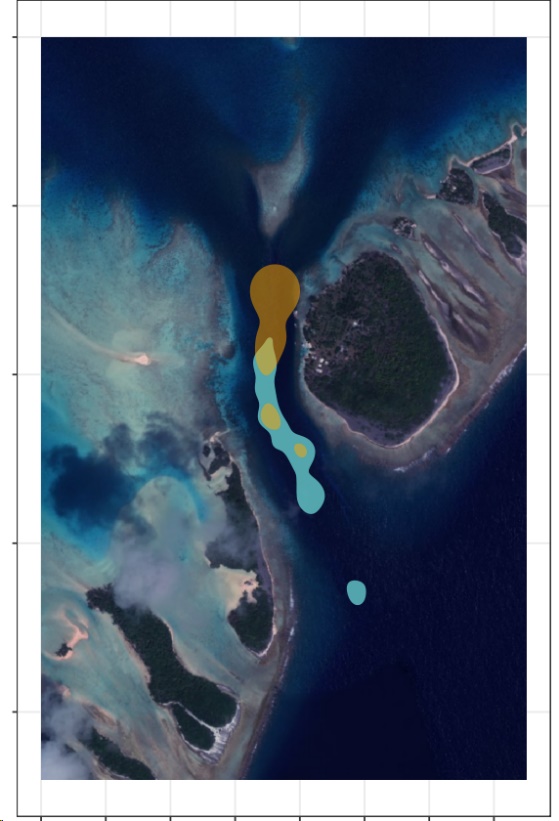
 **DIN DOUT**


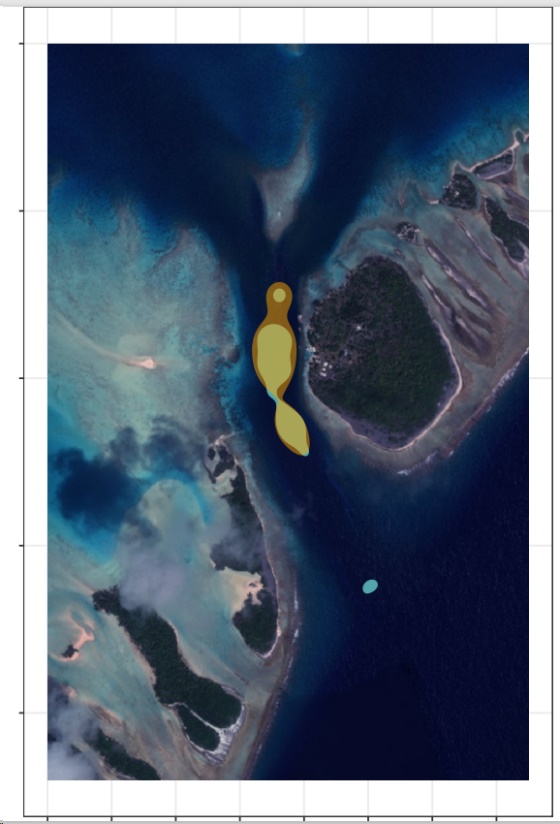


**NIN NOUT**


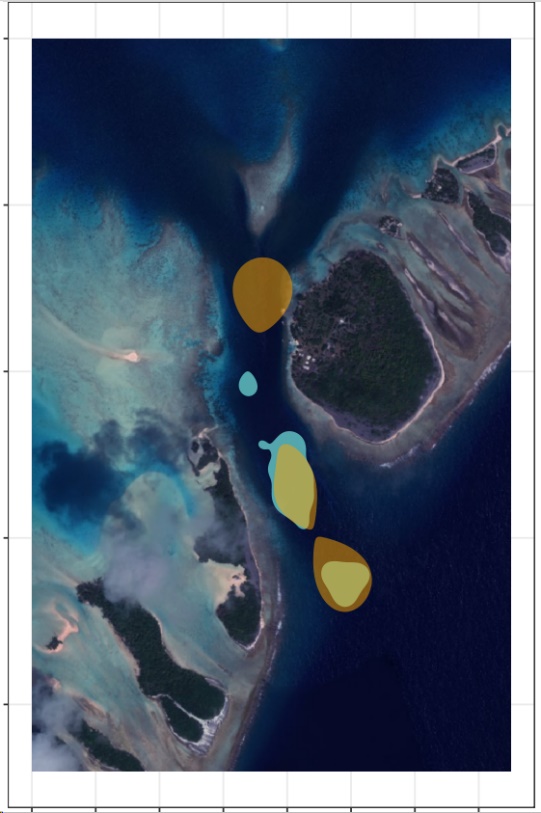

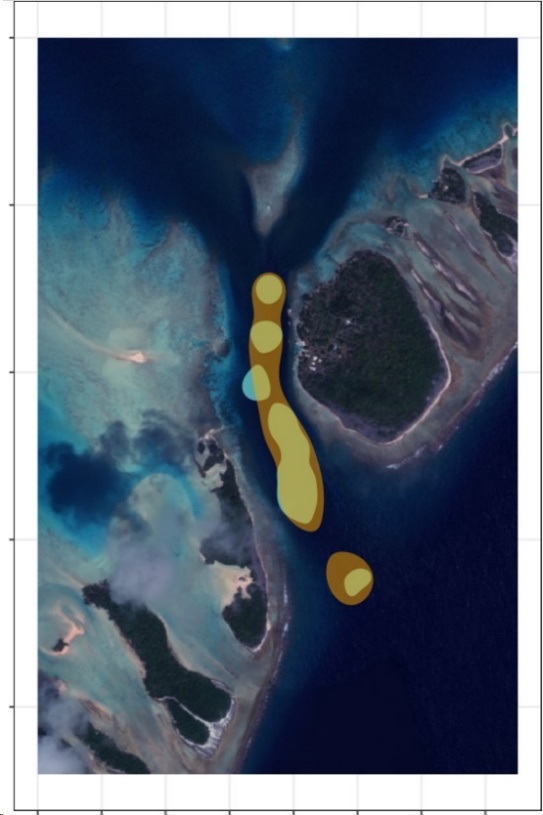


Figure S11: Spatial distribution of regions with high activity values in orange and low activity values in blue without June and July. Upper panels show incoming current and lower panels show outgoing current. Left columns show daytime and right columns show nighttime. Please see Figure in main manuscript for coordinates.


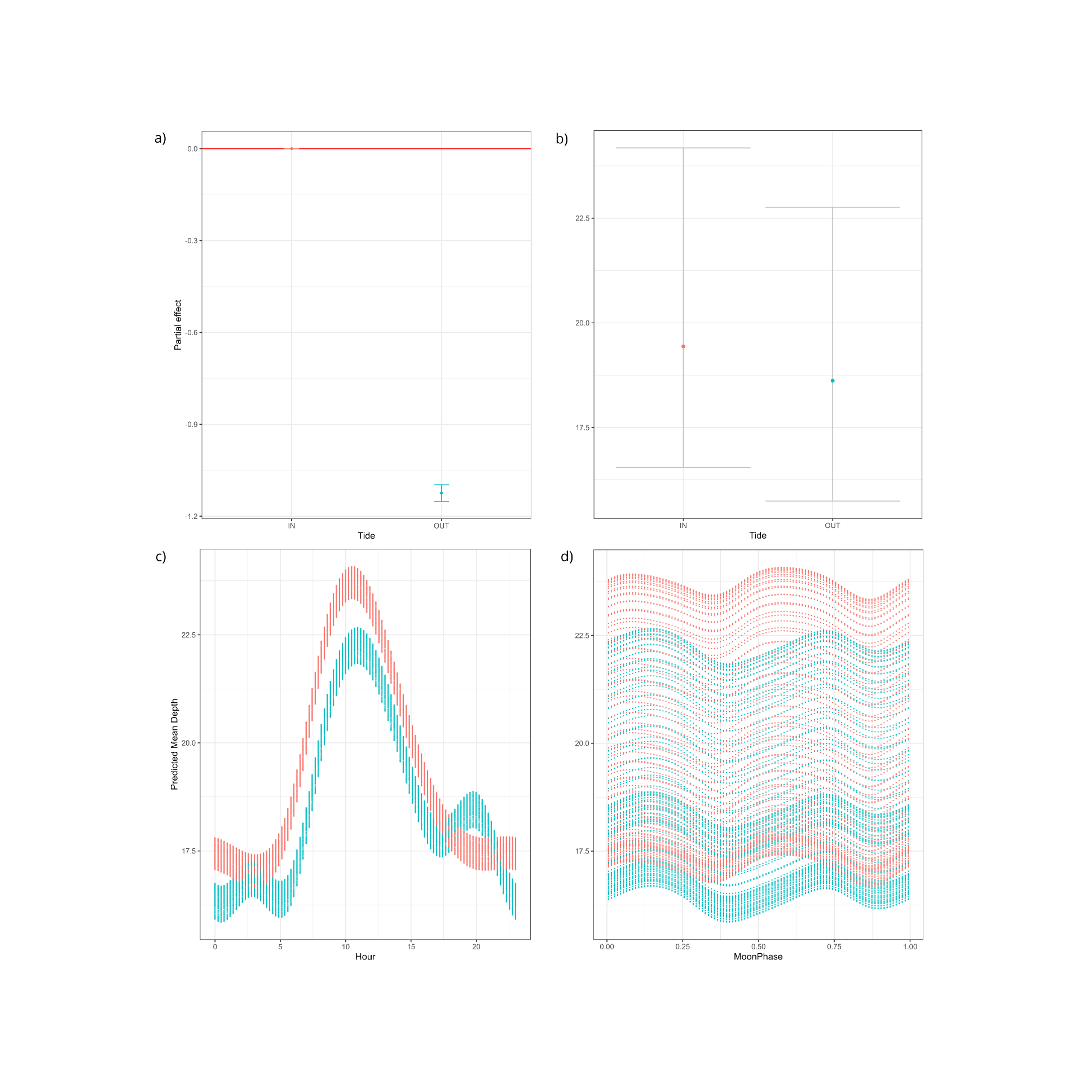


Figure S12: Depth model results. a) The influence of tides on swimming depth; b) The mean of all predicted depth values and the minimum and maximum standard error among all predicted values; c) Predicted depth values according to hours and tides; d) Predicted depth values according to moon phases.

*
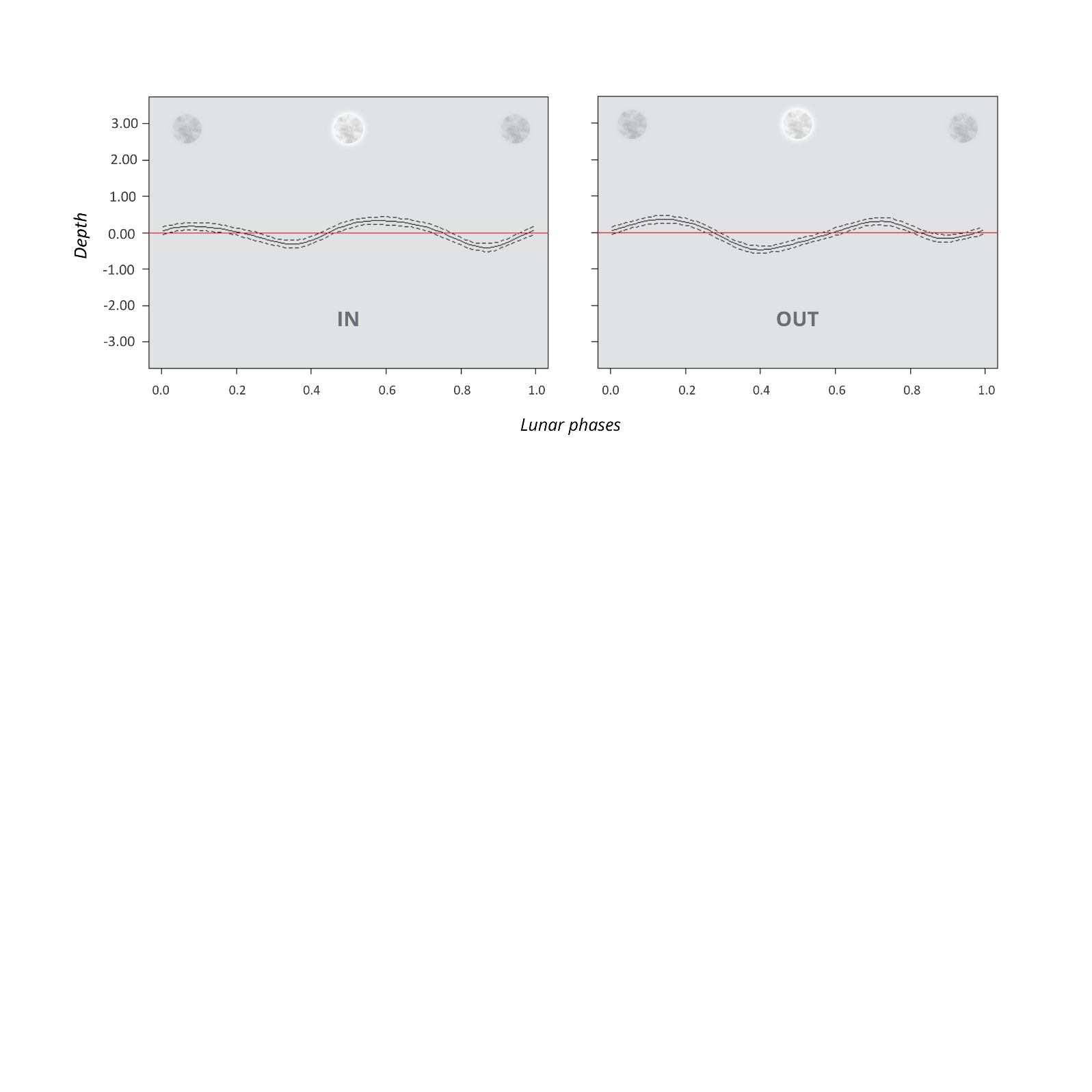
*

Figure S13: Influence of lunar cycles on depth use by *C. amblyrhynchos*. Partial effect of lunar phases and tidal cycle on swimming depth. Depth ~ s(Hour*Tide) + s(Moon*Tide) + Tide + (1|ID) + (1|Month).


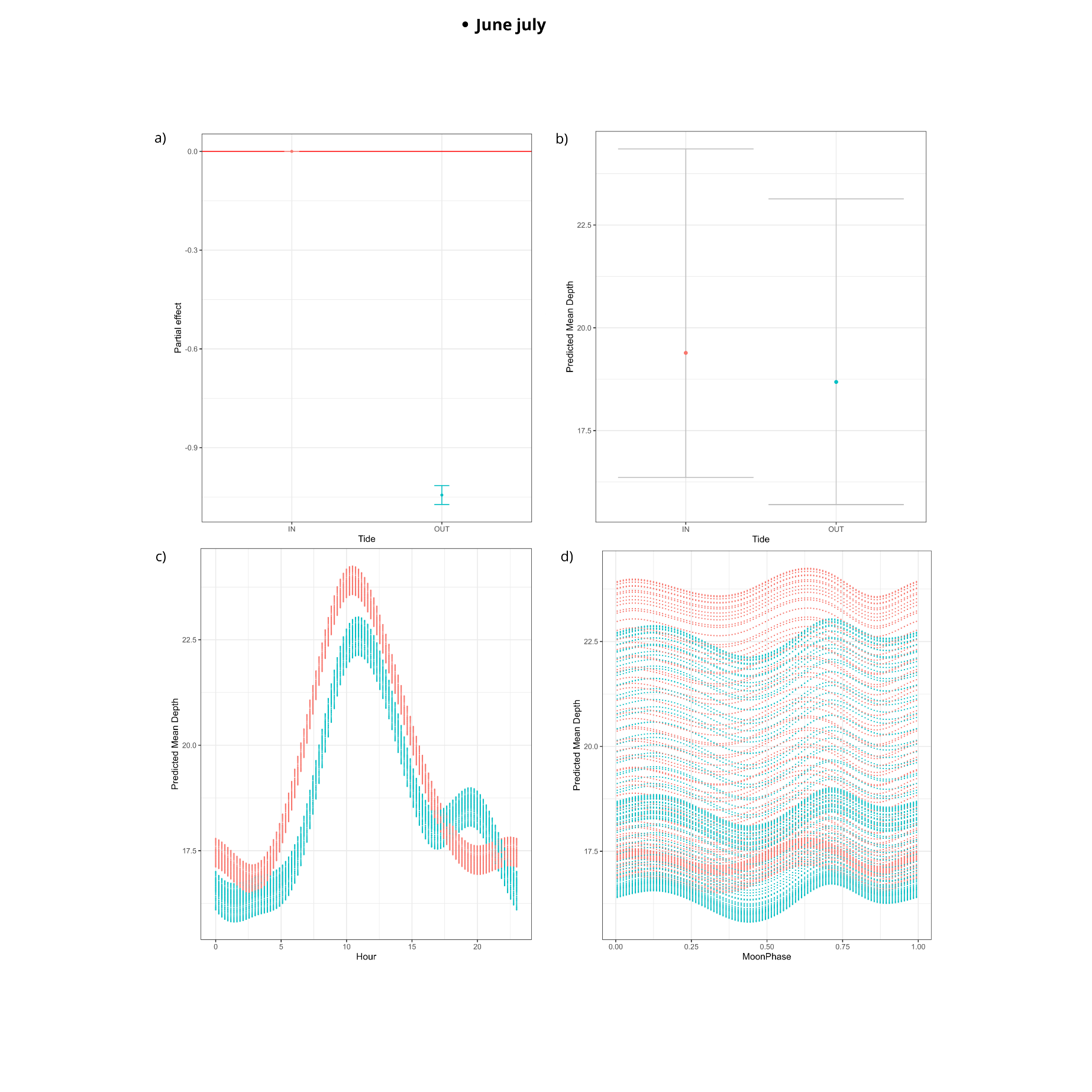


Figure S14: Depth model results without June and July. a) The influence of tides on swimming depth; b) The mean of all predicted depth values and the minimum and maximum standard error among all predicted values; c) Predicted depth values according to hours and tides; d) Predicted depth values according to moon phases

Table S4: Summary of generalized linear mixed models estimating the influence of total length of sharks on a) 50% KUDs and b) 95% KUDs. df: degree of freedom; AIC: Akaike’s information criterion corrected for small sample size; ΔAIC_C_: difference in AIC between the current and top-ranked model; *w*AIC_c_: model probability; R_m_: marginal R² (fixed effects); R_c_: conditional R² (fixed and random effects). Total length was selected only for the 95% KUD model. However, the effect of total length was weak.

| Model | df | logLik | AIC_c_ | ΔAIC_c_ | Waic_c_ | R_m (%)_ | R_c_  _(%)_ |
| --- | --- | --- | --- | --- | --- | --- | --- |
| 1. 50% KUD_log_ |  |  |  |  |  |  |  |
| ~ *Null (1\|ID) + (1\|Month)* | **4** | **-1463.9** | **2935.9** | **0.0** | **1.000** | **0.00** | **36.58** |
| ~ TL + (1\|ID) + (1\|Month) | 5 | -1468.0 | 2946.0 | 10.1 | 0.000 | 0.62 | 37.21 |
| *~ (1\|ID)* | 3 | -1498.5 | 3003.1 | 67.2 | 0.000 | 0.00 | 34.50 |
| *~ (1\|Month)* | 3 | -2095.5 | 4197.0 | 1261.1 | 0.000 | 0.00 | 1.88 |
|  |  |  |  |  |  |  |  |
| b) 95% KUD |  |  |  |  |  |  |  |
| ~ TL + (1\|ID) + (1\|Month) | **5** | **-38055.3** | **76120.6** | **0.0** | **1.000** | **0.81** | **47.36** |
| *~ Null (1\|ID) + (1\|Month)* | 4 | -38063.1 | 76134.3 | 13.6 | 0.000 | 0.00 | 46.69 |
| *~ (1\|ID)* | 3 | -38113.7 | 76233.3 | 112.7 | 0.000 | 0.00 | 44.42 |
| *~ (1\|Month)* | 3 | -38958.2 | 77922.4 | 1801.7 | 0.000 | 0.00 | 2.11 |

Table S5: Effect of total length (TL) on 95% KUD following the top-model selected in Table S3 (95% KUD ~ TL + (1|ID) + (1|Month)). Total length has no effect on 95% KUD.

|  | **Estimate** | **Std.Error** | **df** | **t-value** | **P-value** |
| --- | --- | --- | --- | --- | --- |
| (Intercept) | 248 084.9 | 101 127.2 | 35 | 2.453 | 0.193 * |
| TL | -575.16 | 718.4 | 35 | -0.801 | 0.429 |
